# Subscaling of a cytosolic RNA binding protein governs cell size homeostasis in the multiple fission alga Chlamydomonas

**DOI:** 10.1371/journal.pgen.1010503

**Published:** 2024-03-18

**Authors:** Dianyi Liu, Cristina Lopez-Paz, Yubing Li, Xiaohong Zhuang, James Umen

**Affiliations:** 1 Donald Danforth Plant Science Center, St. Louis, Missouri, United States of America; 2 University of Missouri—St. Louis, Cell and Molecular Biology Program, St. Louis. Missouri, United States of America; Washington University School of Medicine, UNITED STATES

## Abstract

Coordination of growth and division in eukaryotic cells is essential for populations of proliferating cells to maintain size homeostasis, but the underlying mechanisms that govern cell size have only been investigated in a few taxa. The green alga *Chlamydomonas reinhardtii* (Chlamydomonas) proliferates using a multiple fission cell cycle that involves a long G1 phase followed by a rapid series of successive S and M phases (S/M) that produces 2^n^ daughter cells. Two control points show cell-size dependence: the Commitment control point in mid-G1 phase requires the attainment of a minimum size to enable at least one mitotic division during S/M, and the S/M control point where mother cell size governs cell division number (n), ensuring that daughter distributions are uniform. *tny1* mutants pass Commitment at a smaller size than wild type and undergo extra divisions during S/M phase to produce small daughters, indicating that TNY1 functions to inhibit size-dependent cell cycle progression. *TNY1* encodes a cytosolic hnRNP A-related RNA binding protein and is produced once per cell cycle during S/M phase where it is apportioned to daughter cells, and then remains at constant absolute abundance as cells grow, a property known as subscaling. Altering the dosage of *TNY1* in heterozygous diploids or through mis-expression increased Commitment cell size and daughter cell size, indicating that TNY1 is a limiting factor for both size control points. Epistasis placed *TNY1* function upstream of the retinoblastoma tumor suppressor complex (RBC) and one of its regulators, Cyclin-Dependent Kinase G1 (CDKG1). Moreover, CDKG1 protein and mRNA were found to over-accumulate in *tny1* cells suggesting that CDKG1 may be a direct target of repression by TNY1. Our data expand the potential roles of subscaling proteins outside the nucleus and imply a control mechanism that ties TNY1 accumulation to pre-division mother cell size.

## Author summary

Size control is a fundamental property of cells which requires balancing cell growth with cell division, but the mechanisms used by cells to achieve this balance are only partly understood. The best-characterized mechanisms for size control to date involve fixed amounts of nuclear-DNA-bound inhibitory factors which repress cell division until cells grow past a minimum size threshold to overcome the inhibition. The unicellular green alga Chlamydomonas and many other algae and protists use a non-canonical cell cycle where cells can grow by many-fold in size before dividing, and then undergo multiple fission which involves successive rapid divisions to produce a uniform-sized population of daughters. In Chlamydomonas an unknown size homeostasis mechanism couples mother cell size to division number such that larger mother cells divide more times than smaller mother cells. Here, we identified and characterized a key factor governing size control in Chlamydomonas, a cytoplasmic RNA-binding protein and division inhibitor, TNY1, that is produced in a fixed amount in daughter cells and does not increase with cell growth, a property called subscaling. We found that TNY1 represses production of a cell cycle activator, CDKG1, during multiple fission to control daughter cell size. TNY1 is the first example of a cytosolic cell cycle inhibitor that does not depend on nuclear DNA binding to govern subscaling.

## Introduction

Size homeostasis is a fundamental property of proliferating cells and is achieved through mechanisms that balance cell growth with cell division. However, how cells sense and control size remain unexplored in most eukaryotic lineages. Active size control mechanisms have been characterized in several eukaryotes including budding yeast, mammalian tissue culture cells, and Arabidopsis meristems [1–3]. In each case, a titration mechanism operates where a cell cycle inhibitor is produced at a fixed absolute amount per cell in each cell cycle, a property known as subscaling, while an activator accumulates as cells grow [1,4]. At their critical size, cells have accumulated enough activator to overcome the inhibitor and allow cell cycle progression. The details of which proteins acts as the inhibitor or the activator differ in each species, but there are some systems-level similarities in several taxa including G1-S control with a nuclear-localized and/or chromatin associated factor as the subscaling inhibitor [5,6]. Chromatin or nuclear DNA content is a naturally subscaling component of cells that has been exploited in Arabidopsis to ensure that the absolute amount of the inhibitor protein KRP4 apportioned to daughters is independent of birth size [3,4]. In yeast and mammalian cells, chromatin-bound cell cycle inhibitor proteins, Whi5 and Rb respectively, are also subscaling and have been hypothesized to act as limiting inhibitors of cell cycle progression [7,8].

The unicellular green alga *Chlamydomonas reinhardtii* (Chlamydomonas) is a microbial model for plant cell cycles and for non-canonical multiple fission cell cycles that are used by many algae and other protists [9,10]. Multiple fission cell cycles partially uncouple cell growth and cell division: during a prolonged G1 phase, cells can grow more than ten-fold in size. Upon exiting G1, mother cells undergo (*n*) rapid alternating rounds of DNA synthesis and mitosis (S/M) and produce *2*^*n*^ daughters within a common mother cell wall. Upon mitotic exit, the daughters hatch and enter either G0 or G1 phase due to nutrient availability [9,10]. The Chlamydomonas multiple fission cell cycle has two size control points or checkpoints. The Commitment point occurs in G1 phase, and is operationally defined by the transition from growth-dependence to growth-independence for completing at least one cycle of S/M. Cells must reach a minimum size to pass Commitment, and may continue to grow after Commitment for 5–7 hours, but this additional growth is optional for completing at least one cycle of S/M [9–11]. Consequently, mother cells can begin S/M within a very large size range between two and twenty times the modal daughter size [9–11]. A second critical size checkpoint operates during the S/M phase and ensures that larger mother cells divide more times than smaller mother cells so that daughter sizes are in a uniform range regardless of the starting sizes of the mother cell population [9–11]. Thus, multiple fission incorporates a size control mechanism that is conceptually somewhat different than a simple gating mechanism used to control size in binary fission cell cycles.

Previous studies identified mutants that disrupted cell size homeostasis, including mutants affecting each subunit of the Chlamydomonas retinoblastoma tumor suppressor complex (RBC), MAT3/RBR, E2F1, and DP1 [12,13]. Interestingly, both Commitment size and the S/M size checkpoint were changed in these mutants [12,13]. Loss of function mutations in the *MAT3/RBR* gene caused cells to pass Commitment at a smaller size than wild type, and to divide too many times producing small daughters [12]. In contrast, loss of function mutations in the *DP1* gene suppressed the *mat3/rbr* phenotype and caused cells to pass Commitment at a larger size and to divide too few times leading to large daughters [13]. Unlike the proposed model for size control in mammalian cells where the RB protein subscales [7], RBC subunits do not show this subscaling behavior in Chlamydomonas [14,15].

*cdkg1* was isolated in an insertional screen for size control defects. The mutant caused a large daughter cell phenotype and was found to act upstream of the RBC [15]. CDKG1 encodes a D-cyclin dependent kinase (CDK) that phosphorylates the MAT3/RBR subunit of the RBC and is a limiting factor in mitotic size control. While loss of the protein in *cdkg1* mutants caused too few divisions and large cells, over-production of CDKG1 caused extra divisions leading to smaller daughter cells [15]. CDKG1 protein is synthesized just before S/M begins with larger mother cells producing a higher nuclear concentration of CDKG1 than smaller mother cells. Nuclear CDKG1 concentration decreases with each round of cell division. Upon mitotic exit CDKG1 protein becomes undetectable and remains so until the S/M phase of the next cell cycle [15]. It is unknown how *CDKG1* mRNA abundance and CDKG1 protein levels are modulated to control cell division number.

Here, we identified and characterized a Chlamydomonas heterogeneous nuclear ribonucleoprotein (hnRNP) related protein, TNY1, that acts as a cytosolic repressor in the size control pathway upstream of CDKG1 and the RBC. A loss of function mutation in the *TNY1* locus altered Commitment and S/M size control leading to production of small daughters. TNY1 protein was produced once per cell cycle during S/M phase and apportioned to daughter cells where its absolute abundance stayed constant during G1 phase. Gene dosage alteration and mis-expression experiments with *TNY1* both supported its role as a limiting regulator of mitotic size control. At least one key target of TNY1 repression is CDKG1, whose mRNA and protein abundance were negatively regulated by TNY1. TNY1 was found to be part of a ribonucleoprotein complex *in vivo*, and was able to bind the unusually long and uridine-rich 3’ untranslated region of the *CDKG1* mRNA *in vitro*. TNY1 is a novel example of a non-nuclear subscaling inhibitor which governs size control in Chlamydomonas.

## Results

### TNY1 is a negative regulator of cell division upstream of CDKG1

**Fig 1:**
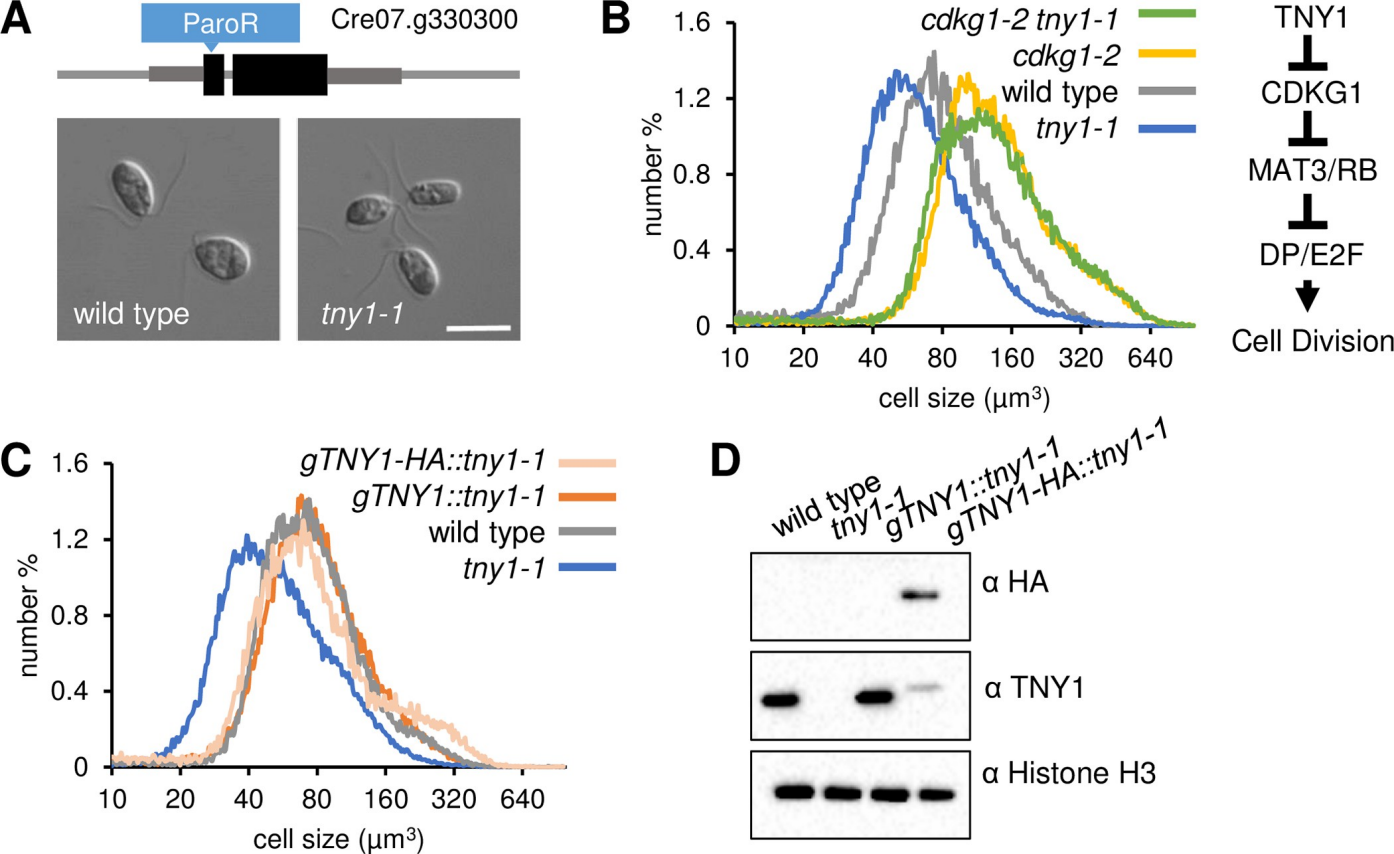
Identification of TNY1 as a regulator of cell size in the retinoblastoma pathway. (A) Upper panel, schematic of *TNY1* locus with location of an inserted paromomycin resistance marker (paroR in blue) in exon 1 that produced the *tny1-1* allele. Black rectangles, exons; dark gray rectangles, untranslated regions; narrow gray lines, introns and intergenic regions. Lower panel, Differential Interference Contrast (DIC) images of daughter cells from wild type parent strain CC-124 and *tny1-1*. Scale bar = 10 μm. (B) Left panel, size distributions of daughter cells from *tny1-1* (median size 44 μm^3^/modal size 40 μm^3^), wild type CC-124 (median size 66 μm^3^/modal size 70 μm^3^), *cdkg1-2* (median size 117 μm^3^/modal size 113 μm^3^), and *cdkg1-2 tny1-1* (median size 113 μm^3^/modal size 117 μm^3^). Median size of wild type > *tny1-1* (p<0.01, Student’s t-test) (S1 Table). Median sizes of *tny1-1* and *tny1-1 cdkg1-2* are not different (p>0.1, Student’s t-test) (S1 Table). Right panel, epistasis diagram showing positive (arrows) and negative (bars) regulators of size-dependent cell division. *TNY1* functions upstream of *CDKG1*. (C) Size distributions of daughter cells from *tny1-1* (median size 44 μm^3^/modal size 40 μm^3^), wild type CC-124 (median size 69 μm^3^/modal size 74 μm^3^), *tny1-1* rescued strains *gTNY1*::*tny1-1* (median size 72 μm^3^/modal size 75 μm^3^) and *gTNY1-HA*::*tny1-1* (median size 66 μm^3^/modal size 70 μm^3^). Median sizes of daughter cells of wild type, *gTNY1*::*tny1-1*, and *gTNY1-HA*::*tny1-1* are not significantly different (p>0.1) by one way ANOVA testing (S1 Table). (D) Immunoblots of SDS PAGE separated protein lysates from daughter cells of indicated genotypes using α-HA, α-TNY1, or α-histone H3 (internal loading control).

*tny1-1* mutants were discovered in a forward insertional mutagenesis screen using a paromomycin antibiotic selection marker (paroR) with direct screening for size defects of plate-grown gametes using a Coulter Counter. *tny1-1* gametes arrested in early G1 phase showed a small size phenotype and the mutant was re-tested under more controlled vegetative growth conditions to assess daughter cell size (Fig 1A). Wild type parental strain CC-124 and *tny1-1* cultures were synchronized under a diurnal cycle and daughter cell sizes were measured. *tny1-1* daughter cells had a modal cell size of ~45 μm^3^ compared with ~75 μm^3^ for wild type daughters (Fig 1B and S1 Table), with both strains passing Commitment and entering S/M with similar timing (S1A Fig), though with *tny1-1* populations always at a smaller size than the control population when undergoing these two transitions (S1B Fig). The time interval between Commitment and entering S/M was the same in wild type and *tny1-1* mutants, so the small size defects of *tny1-1* strains are not attributable to a shortened cell cycle duration (S1A and S1B Fig). We next generated populations of wild type and *tny1-1* mother cells and compared cell division numbers using a Commitment assay (Methods). Synchronized *tny1-1* and wild-type strains were sampled at the same time points in mid- or late-G1 phase and had similar division number profiles (S1C Fig), despite the wild-type cells mother cells being 50% to 60% larger (S1B Fig). In experiments where mother cell size distributions were matched between the two strains (modal size ~230 μm^3^), *tny1-1* mother cells underwent an average of 2.8 rounds of multiple fission versus 1.4 rounds for wild type (S1D Fig).

Besides causing a shift in cell size set points, size control mutants can also increase size distribution variance due to weakened coupling between cell size and division control mechanisms as we recently observed in *mat3/rbr* mutants [11]. The variance of an approximately log-normal distribution found in synchronized Chlamydomonas populations can be described using log transformed size bins which preserve symmetry around the mean of the distribution and are more intuitive to interpret than the same data described in linear space. Using this transform we compared standard deviation (SD) and coefficient of variance (CV) in daughter populations of wild-type, *tny1-1*, *cdkg1*, and *tny1-1* strains expressing rescuing transgenes (S2A Fig). CVs of cell populations are sensitive to small deviations in synchrony and had some variability between replicates, but the best-synchronized populations of *tny1-1* and *cdkg1-2* daughter cells had CVs similar to wild type controls (S2 Fig). This finding suggests that TNY1 is needed for establishing the relationship between mother cell size and division number during S/M, but that in its absence separate or redundant mechanisms govern the strength of coupling between cell size and division behavior. In summary, the overall timing of cell cycle events is normal in *tny1-1* mutants, but the minimum Commitment cell size and S/M phase size control of *tny1-1* cells are both mis-regulated in a manner consistent with TNY1 acting as a negative regulator for size-dependent cell cycle control points.

We next used epistasis experiments to determine the relationship of *tny1-1* to other cell size regulators. CDKG1 functions upstream of the RBC, and *cdkg1-2* null mutants cause a large-cell phenotype [15]. *cdkg1-2 tny1-1* double mutants had nearly identical sizes as *cdkg1-2* single mutants indicating that TNY1 functions upstream of CDKG1 and the RBC and does not appear to control cell size homeostasis through an independent mechanism (Fig 1B and S1 Table). Commitment sizes for *cdkg1-2* and *cdkg1-2 tny1-1* (~200 μm^3^) are very similar to the Commitment size (~200 μm^3^) of a wild-type strain (S3A and S3B Fig), indicating that *cdkg1-2* suppresses both the Commitment and the S/M size defects of *tny1-1*.

The *tny1-1* strain was found to contain a single insertion of the paromomycin (paroR) marker in the first exon of Cre07.g330300 [16] (Fig 1A). *tny1-1* was back-crossed to wild-type strain CC-125 and random progeny were selected and scored for gamete cell size, mating type, and paromomycin resistance (paroR) or sensitivity (ParoS). The paroR segregants produced small gametes, while the paroS segregants were wild-type size indicating linkage between the paromomycin cassette insertion and the *tny1-1* phenotype (Methods, S3C–S3E Fig). Rescue of the *tny1-1* small cell defect was performed by transforming constructs that contained either a full-length genomic fragment of wild type Cre07.g330300 (gTNY1) or a version with a C-terminal triple hemagglutinin epitope tag (gTNY1-3xHA). In both cases, normal daughter cell sizes were restored in a fraction of transformants while no rescue was observed in control transformants bearing an empty vector (Fig 1C and S1 Table). Rescue efficiency with either of the two constructs was somewhat low (~2%) but not atypical for Chlamydomonas rescues. Immunoblotting of SDS-PAGE separated proteins from wild type, *tny1-1*, and rescued *tny1-1* strains using polyclonal antibodies raised against recombinant TNY1 protein or α-HA antibodies detected proteins of the expected migration (~48 kDa) in wild type and rescued strains showing that TNY1 expression was restored in those rescued lines (Fig 1D). Together these experiments confirm that disruption of Cre07.g330300 causes the *tny1-1* phenotype.

### *TNY1* is predicted to encode a putative hnRNP A-related RNA binding protein

**Fig 2:**
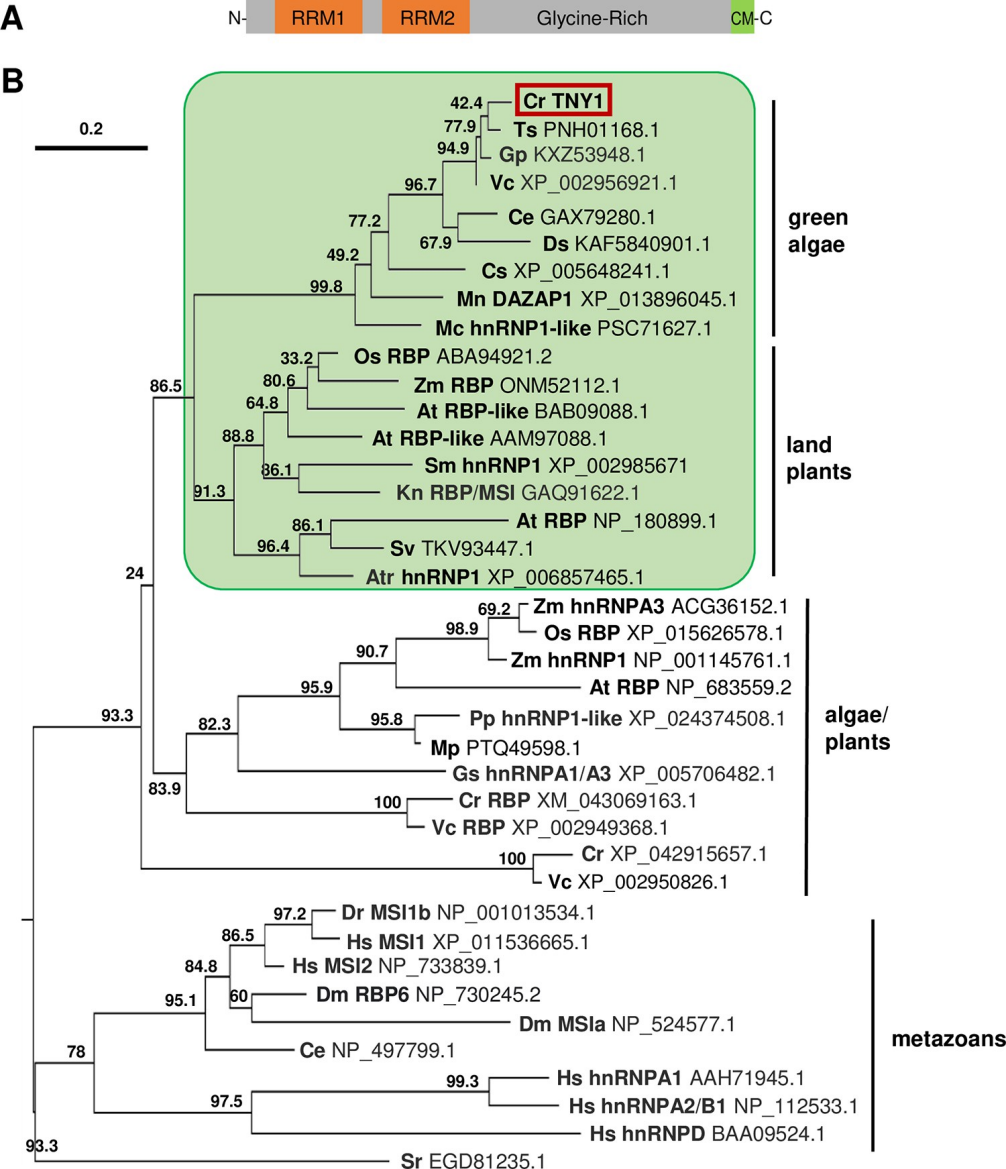
*TNY1* encodes a hnRNP-related RNA binding protein. (A) Schematic of predicted TNY1 protein domain structure from N to C terminus. Two RNA binding motifs (RRM1 and RRM2, orange bars) are followed by a glycine-rich region and a short, conserved motif (CM) at the C-terminus. (B) Maximum likelihood phylogeny TNY1 and related hnRNP related proteins in indicated taxonomic groups. Species abbreviations are followed by protein names and NCBI protein IDs. Cr, *Chlamydomonas reinhardtii*. Ts, *Tetrabaena socialis*. Gp, *Gonium pectorale*. Vc, *Volvox carteri*. Ce, *Chlamydomonas eustigma*. Ds, *Dunaliella salina*. Cs, *Coccomyxa subellipsoidea*. Mn, *Monoraphidium neglectum*. Mc, *Micractinium conductrix*. Os, *Oryza sativa*. Zm, *Zea mays*. At, *Arabidopsis thaliana*. Sm, *Selaginella moellendorffii*. Kn, *Klebsormidium nitens*. Sv, *Setaria viridis*. Atr, *Amborella trichopoda*. Pp, *Physcomitrella patens*. Mp, *Marchantia polymorpha*. Gs, *Galdieria sulphuraria*. Dr, *Danio rerio*. Dm, *Drosophila melanogaster*. Ce, *Caenorhabditis elegans*. Hs, *Homo sapiens*. Sr, *Salpingoeca rosetta*.

*TNY1* is predicted to encode a protein with two N-terminal RNA recognition motifs (RRMs) and a low complexity glycine-rich C-terminus (Figs 2A and S4). This structure is found in eukaryotic heterogeneous nuclear ribonucleoproteins (hnRNPs) and other related RNA binding proteins that have diverse roles in nucleic acid regulation and metabolism, functioning as RNA or DNA binding proteins [17,18]. BLAST searching in different taxa was used to identify proteins related to TNY1 in animals, plants, and algae. These sequences were curated and used to estimate a maximum likelihood phylogeny which placed TNY1 in a clade of green algal TNY1-like homologs, and this TNY1 clade was sister to a larger grouping of plant tandem RRM hnRNP-like proteins suggesting a common origin at the base of the Viridiplantae (Methods, Fig 2B). While Chlamydomonas encodes other hnRNP-like proteins, these are grouped outside of the green algal TNY1 clade which may have originated in the crown Chlorophytes (Chlorophyceae/Trebouxiophyceae/Ulvophyceae). No close matches to TNY1 were found in predicted proteomes of earlier diverging prasinophycean grade Chlorophytes including *Micromonas* and *Ostreococcus* which both have reduced genomes and may have lost ancestral *TNY1*-related genes.

### TNY1 is localized in the cytosol

**Fig 3:**
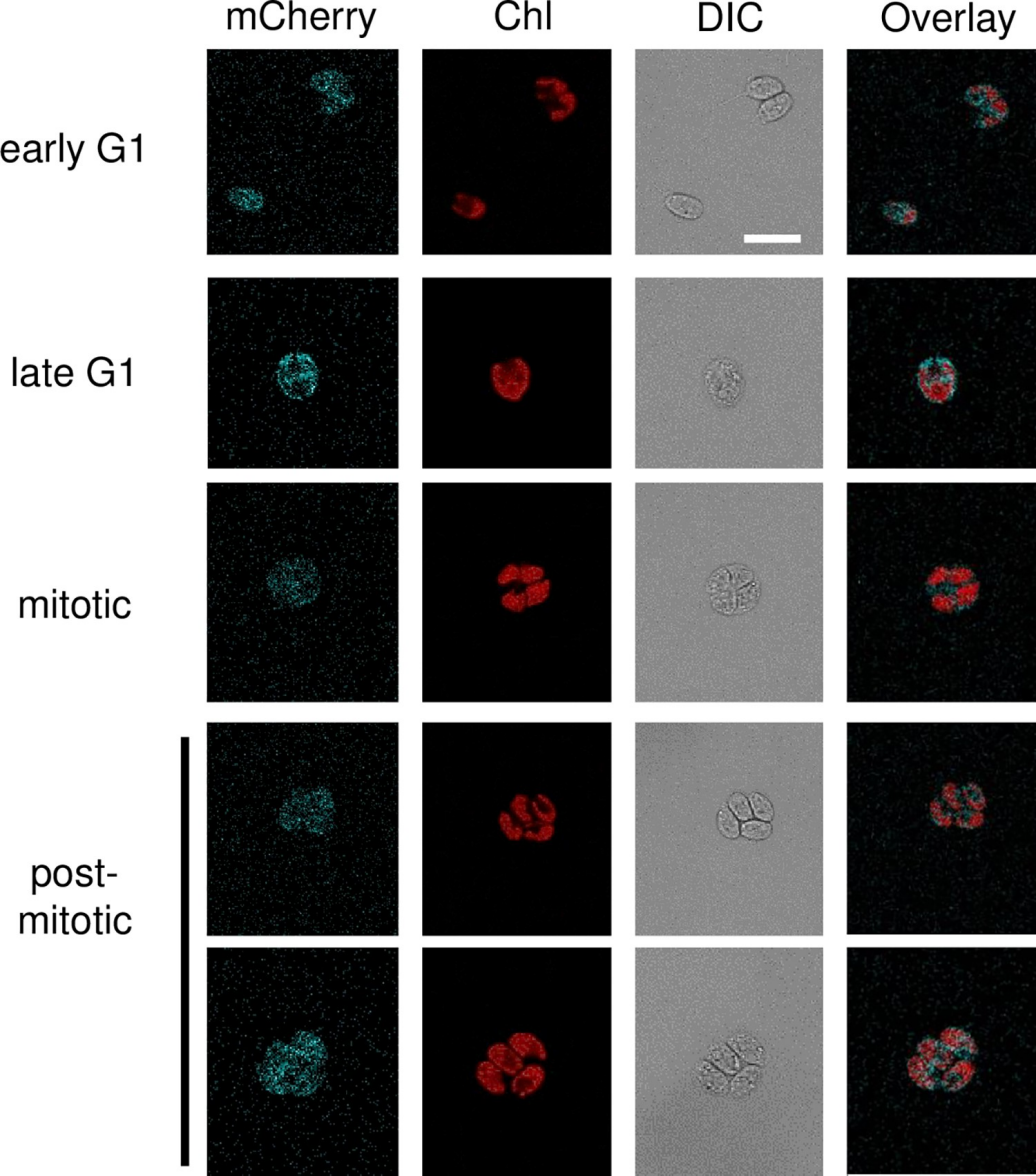
TNY1 is localized in the cytosol. Confocal fluorescence images of live cells at different cell cycle phases (left side labels) expressing a functional TNY1-mCherry fusion protein. TNY1-mCherry signal (mCherry, pseudo colored cyan). Chlorophyll fluorescence (Chl, pseudo colored red). Differential Interference Contrast (DIC). Merged fluorescent images (Overlay). Scale bar = 10 μm.

To determine the subcellular localization of TNY1, a genomic *TNY1* construct with a C-terminal fusion of Chlamydomonas codon-optimized *mCherry* was used to rescue *tny1-1* mutants and generate *gTNY1-mCherry*::*tny1-1* strains with fusion protein expression detected by immunoblotting (S5A Fig), and confirmed with a rescued size phenotype (S5B Fig and S1 Table). Live cell confocal fluorescence microscopy revealed TNY1-mCherry signal in the cytosol throughout the vegetative cell cycle (Figs 3 and S5C). Indirect immunofluorescence using α-HA antibodies targeting tagged TNY1-HA confirmed the cytosolic location and showed exclusion of TNY1 protein signal from the nucleus (S5D Fig).

### TNY1 regulation and subscaling throughout the cell cycle

**Fig 4:**
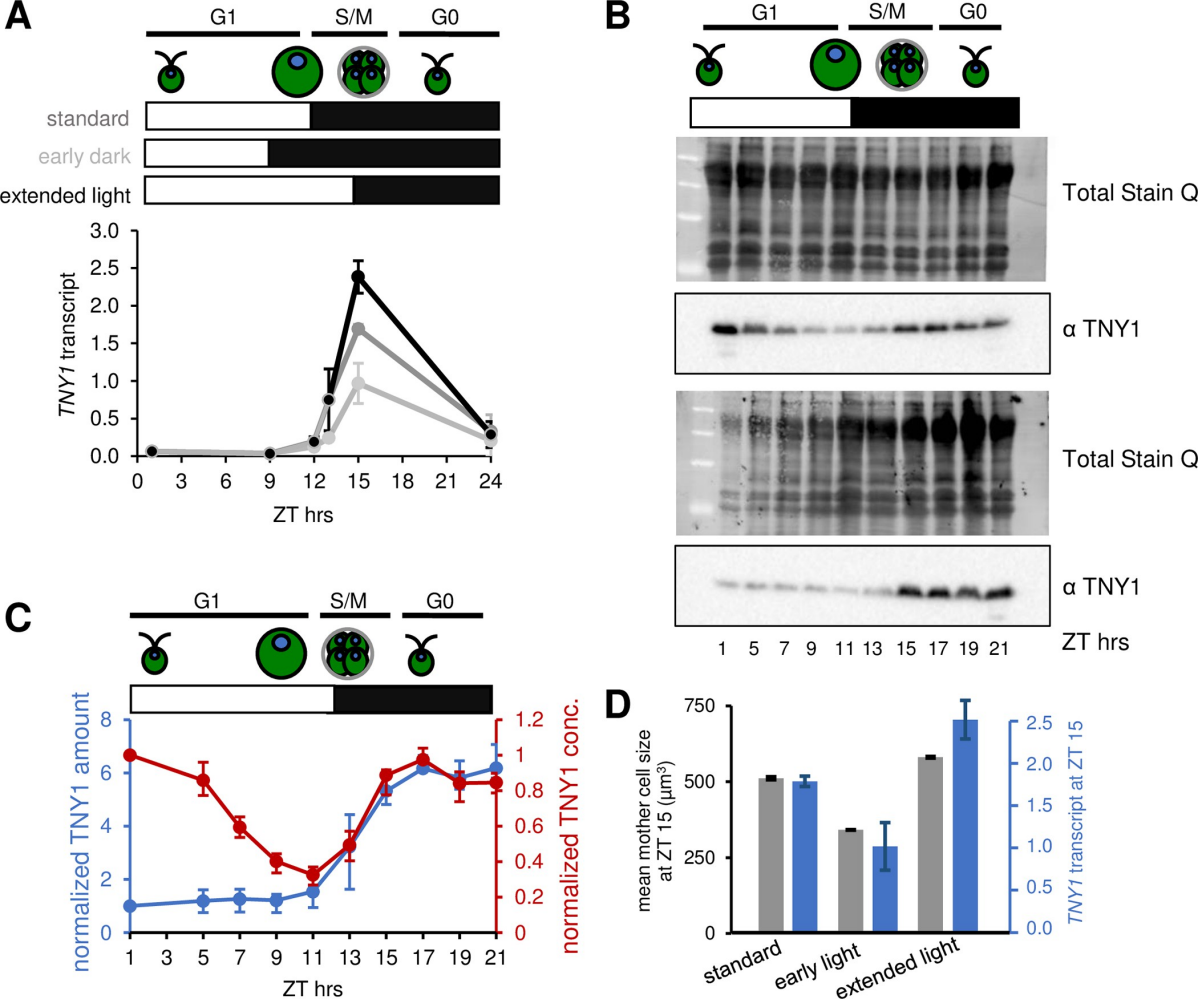
Cell cycle control of *TNY1* mRNA and TNY1 protein accumulation. (A) qRT-PCR data time series for *TNY1* mRNA accumulation in synchronous wild type cultures with light phase (white bar) and dark phase (black bar), and cell cycle phasing cartooned above. Cultures were synchronized under a standard diurnal regime (dark grey line, 12hr:12hr light:dark), in parallel with two modified diurnal regimes of early dark (light grey line, 15hr:9hr light:dark) or extended light (black line, 9hr:15hr light:dark). Under all three different diurnal regimes >80% of cells divide between ZT 12 hrs ZT 15 hrs. *TNY1* transcripts were normalized against an internal control *GBLP* transcripts (Methods) and plotted as the average and SD (error bars) of three biological replicates. (B) Representative immunoblots with whole cell lysates from synchronized wild-type cultures under a 12hr:12hr light:dark regime with sampling at indicated time points. Each image set shows total protein signal (top) and α TNY1 signal (bottom). Upper set was loaded with equal protein in each lane, and lower set with equal culture volume per lane (with equal cell number in G1 samples). (C) Plot of TNY1 abundance across the cell cycle to show TNY1 concentration (red curve) or amount per cell (blue curve) in arbitrary units from three biological replicates. Error bars: SD of the average of three biological replicates. (D) Mean mother cells size (grey) and *TNY1* mRNA transcript abundance (blue) for three diurnal regimes shown in panel (A) at ZT 15 hrs. Error bars: SD of three biological replicates.

To determine the accumulation pattern of *TNY1* mRNA during the cell cycle, wild-type cultures were synchronized under a standard diurnal cycle (12hr:12hr light:dark) and RNA samples were prepared from cells at different time points and used for quantitative RT-PCR. *TNY1* mRNA was present at very low levels during G1 phase and rose sharply to a peak toward the middle/end of S/M phase, and then declined in the dark phase after division (Fig 4A). This experiment largely reproduced the results of previous genome-wide expression studies [19,20], where the timing of *TNY1* mRNA accumulation coincided with that of many late mitotic and cilia-related genes.

The trigger for *TNY1* mRNA accumulation is likely to be entry to S/M phase, but we could not rule out diurnal control or the light-to-dark transition as signals for *TNY1* expression. To distinguish these possibilities, we used two alternative diurnal regimes where the light-to-dark transition was shifted forward or backward by three hours, but the timing of S/M phase [15] and *TNY1* peak expression were unaffected (Fig 4A).

The accumulation pattern of TNY1 protein throughout the cell cycle was determined by quantitative immunoblotting of samples taken from wild-type cultures synchronized under the standard 12hr:12hr light:dark diurnal regime (S6 Fig). Samples were loaded either by equal protein per lane which reflects TNY1 concentration in cells (Figs 4B and S7A–S7E, upper blots) or by equal culture volume per lane which reflects amount of TNY1 per cell in G1 phase samples (Figs 4B and S7A–S7E, lower blots). Plots of TNY1 signal during the cell cycle (Figs 4C and S7F and S2 Data) showed a constant amount per cell during G1 phase as cells increased in size by around six-fold, and an increase during S/M phase as cells divided. The complementary curve of TNY1 concentration shows it is highest in early G1 daughter cells, and drops as cells grow during G1 phase, and restored at cell division. In summary, cells are born with a fixed amount of TNY1 protein that is steadily diluted during G1 phase as cells grow, reaching its minimum concentration just prior to S/M during which its mRNA is transcribed and the protein is replenished in new daughters (Figs 4C and S7F and S2 Data).

We next determined how *TNY1* gene expression scaled with mother cell size during S/M phase. We compared mean mother cell size in samples from the three regimes in Fig 4A (S7G Fig) to the *TNY1* mRNA expression peak height and found they are correlated, suggesting that mother cell size or numbers of daughter nuclei may control *TNY1* mRNA production (Fig 4D).

### TNY1 is limiting for size control

**Fig 5:**
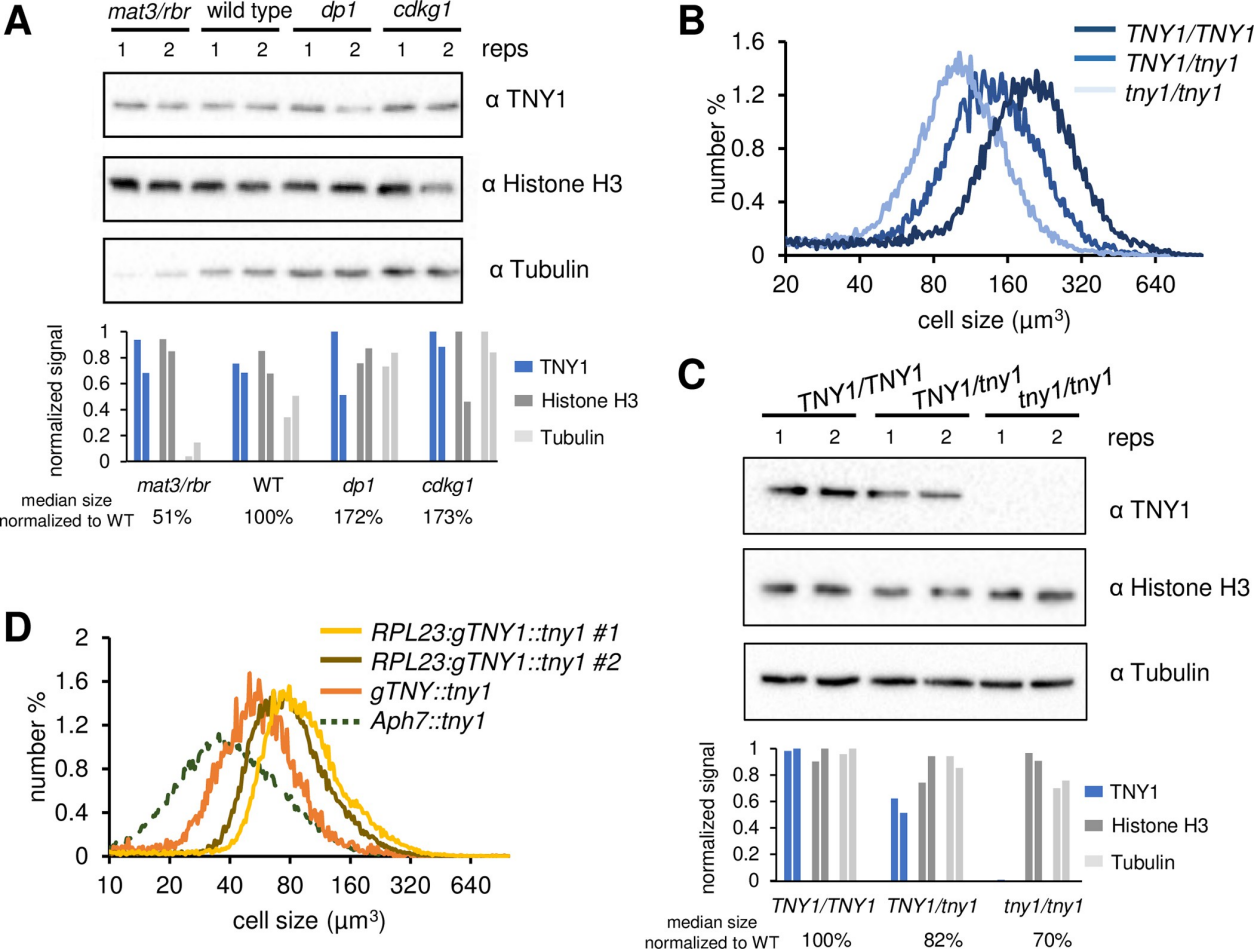
TNY1 is limiting in cell size control. (A) Immunoblots with whole cell lysates from wild type or mutant daughter cells (ZT 0hr under standard conditions) with two replicates each (reps 1 and 2). Each gel was probed with α-TNY1, α-Histone H3 and α-Tubulin. Bar graphs show signals in arbitrary units for each replicate band with the strongest band in each blot set to 1. (B) Size distributions of diploid daughter cells of indicated genotypes. *tny1/tny1* (median size 111 μm^3^/modal size 96 μm^3^), *TNY1/tny1* (median size 124 μm^3^/modal size 113 μm^3^), and *TNY1/TNY1* (median size 154 μm^3^/modal size 166 μm^3^). Median size of *TNY1/TNY1* > *TNY1/tny1* (p< 0.01, one-tailed t-test). Median size of *TNY1/tny1* > *tny1/tny1* (p< 0.01, one-tailed t-test) (S1 Table). (C) Immunoblots with whole cell lysates from indicated diploid daughter cells with two replicates each (reps 1 and 2). Immunoblots were loaded and processed similar to those in Fig 5A. Quantitation of the immunoblot signals were plotted below as described in panel A. (D) Size distributions of synchronous daughter cells of two independent *RPL23*:*TNY1*::*tny1-1* rescued strains (#1 median size 98 μm^3^/modal size 98 μm^3^ and #2 median size 89 μm^3^/modal size 95 μm^3^), a control strain transformed with resistance marker only *Aph7*:*tny1-1* (median size 44 μm^3^/modal size 40 μm^3^), and a *gTNY1*::*tny1-1* rescued strain (median size 74 μm^3^/modal size 80 μm^3^). Median sizes of four independent *RPL23*:*TNY1*::*tny1* transformants > wild type (p< 0.05, one-tailed t-test) (S1 Table).

To determine if subscaling of TNY1 is controlled by feedback from size control regulators, we examined its levels in cell size mutants. TNY1 protein levels were determined in dark-shifted daughter populations (equivalent to ZT 0 hrs in our light:dark regime) produced from wild type, *mat3-4/rbr*, *dp1-1* and *cdkg1-2* cells (S8A Fig and S1 Table). Interestingly, daughter populations with different mean sizes contained the same amount of TNY1 on a per cell basis suggesting that subscaling of TNY1 is independent of daughter cell size and its levels may instead be controlled by limiting factors that scale invariantly with cell size such as genomic template for *TNY1* transcription (Fig 5A and S8B Fig). If so, then TNY1 abundance may be sensitive to gene dosage. To test gene dosage effects, we created a set of isogenic diploid strains with genotypes *TNY1*/*TNY1*, *TNY1*/*tny1-1*, and *tny1-1*/*tny1-1* (Methods). Size profiles of daughters from synchronized diploid cultures of each strain were compared and found to differ based on TNY1 dosage, with heterozygote daughter size in between that of wild type and homozygous mutants (Fig 5B and S1 Table). TNY1 protein abundance in daughter cells of *TNY1/tny1* heterozygous daughters was also reduced compared with homozygous *TNY1/TNY1* strains (Fig 5C). We also examined cell sizes from haploid meiotic progeny of crosses between *tny1-1*::*TNY1* (or *tny1-1*::*TNY1-HA*) and wild type where progeny could inherit, 0, 1 or 2 copies of *TNY1*. As with the diploid dosage series, the progeny that inherited two copies of *TNY1* were larger than progeny with a single copy (S8C Fig). Besides altering gene dosage, we also generated a *TNY1* transgene driven by a constitutive promoter/terminator from the Chlamydomonas *RPL23* gene [21]. This *RPL23*:*gTNY1*:*RPL23* construct was transformed into a *tny1-1* strain and transformants were tested for size phenotypes along with control transformants that received an empty vector [22]. Among 24 independent *RPL23*:*gTNY1*::*tny1-1* transformants, ~ 20% showed a large daughter cell phenotype with a modal cell size > 80 μm^3^ that was never observed in controls or wild-type rescues (Fig 5D). The large-cell transformants appeared to progress through the cell cycle with similar kinetics as wild type and *tny1* mutants but were larger at each transition (S8D and S8E Fig). Taken together, these data indicate that dosage and expression level of TNY1 impact mitotic cell size control and are consistent with the subscaling behavior observed for TNY1 expression being an important contributor to size-dependent cell cycle control.

### TNY1 inhibits the accumulation of *CDKG1* mRNA and protein in postmitotic cells

**Fig 6:**
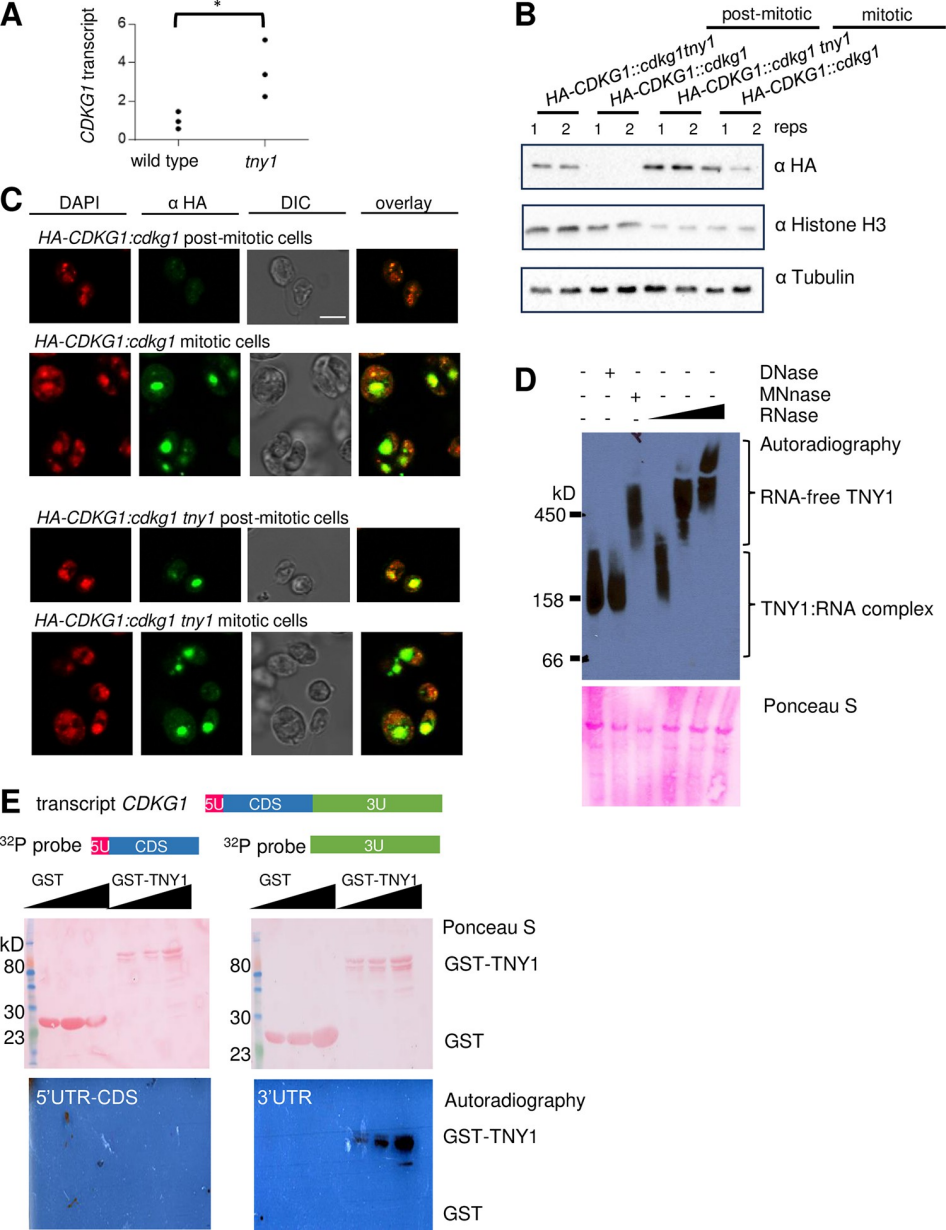
TNY1 inhibits the accumulation of *CDKG1* mRNA and CDKG1 protein. (A) qRT-PCR quantitation of average *CDKG1* mRNA level in daughter cells of wild type and *tny1-1* normalized to internal control gene *GBLP* (Methods). Three biological replicates (averaged value of two technical replicates) are plotted for each genotype with the mean wild-type signal set to 1. Wild-type and *tny1-1* were significantly different by t-test (*, p<0.01). (B) Immunoblots with HA-CDKG1-expressing daughters loaded with equal protein per lane and probed with α-HA, α-Tubulin or α-Histone H3. (C) Brightfield and immunofluorescence microscopy images of representative *HA-CDKG1*::*cdkg1* and *HA-CDKG1*::*cdkg1 tny1* cells. Synchronous mitotic and post-mitotic cells were probed for HA-CDKG1 (α-HA, pseudo-colored green) and stained with DAPI (pseudo-colored red). Note that some of the α-HA pixels were saturated, but all images were taken with identical settings. Scale bar = 10 μm. (D) Native gels were loaded with whole cell lysates from a *gTNY1-HA*::*tny1-1* strain, fractionated and immunoblotted using α-HA. Lysates were pre-treated with different nucleases prior to loading as indicated above each lane. RNase used at different concentrations is indicated by the triangle from lowest to highest 0.01 mg/mL, 0.1mg/mL, and 1mg/mL. The lower image is the same membrane stained with Ponceau S as a protein loading control. (E) Blots containing recombinant GST-TNY1 or GST probed with ^32^P labeled *CDKG1* 3’UTR or *CDKG1* 5’UTR and CDS (Methods). The total protein input was visualized by Ponceau S staining and the ^32^P signal by film-based autoradiography.

Because the *cdkg1* large cell phenotype is epistatic to the *tny1* small cell phenotype we investigated a possible antagonistic relationship between TNY1 and CDKG1 where TNY1 might limit production of CDKG1. In post-mitotic *tny1-1* daughter cells we detected a three-fold increase in *CDKG1* mRNA compared with wild type (Fig 6A). To test the impact of TNY1 on CDKG1 protein abundance *tny1-1* was crossed into a rescued *cdkg1* strain expressing an HA epitope tagged allele *HA-CDGK1*, and expression was assessed by immunoblotting [15]. In mitotic cells we did not consistently see a difference in HA-CDKG1 signal between wild type and *tny1-1* strains, likely due to opposing and non-linear effects of i) mother cell size—which would amplify the CDKG1 signal in wild-type mother cells over the smaller mother cells of *tny1-1* cells (S9A Fig)—and ii) the *tny1-1* mutation—which could increase CDKG1 abundance over what it would have been for a similar-sized wild type cell, but not necessarily over that of the matched control strain with larger mother cells. We instead focused on post-mitotic cells where we consistently observed more HA-CDKG1 in *tny1-1* versus wild-type cells (Figs 6B and S9B). Indirect immunofluorescence (IF) was also used to detect HA-CDKG1 in mitotic and post-mitotic cells [15], where a clear HA-CDKG1 signal was present in around 70% of *tny1-1* daughters (97/145 cells) but never in the *TNY1* control strain (0/133 cells) (Figs 6C and S9C). It is unclear whether the *tny1-1* daughters without a *CDKG1* IF signal were truly negative or below the detection limit of the IF experiment; but the high proportion of HA-CDKG1 positive staining post-mitotic cells in *tny1-1* (but not *TNY1*) strains was reproducible in two independent staining experiments for each genotype. Together these data show that TNY1 limits the accumulation of both *CDKG1* mRNA and CDKG1 protein in post-mitotic cells.

### TNY1 is part of an RNP complex and can bind to the 3’UTR of *CDKG1* mRNA

The finding that cytosolic TNY1 could inhibit accumulation of nuclear-localized CDKG1 protein suggests a mechanism which might involve direct interaction of TNY1 with *CDKG1* mRNA. We first used native electrophoresis of whole cell extracts, and immunoblotting to determine if TNY1 might be part of a ribonucleoprotein complex (RNP). On native gels, TNY1 migrated near the 158 kDa marker, but shifted to a slower migrating complex (>450 kDa) when treated with ribonuclease A (RNAse) or micrococcal nuclease (MNase), but not deoxyribonuclease (DNase). These results suggest that TNY1 is associated with RNA *in vivo* as an RNP, and that the RNA component may contribute significantly to the negative charge state of the complex leading to faster migration when present (Fig 6D).

A simple model for regulation of CDKG1 by TNY1 is direct binding of TNY1 to the *CDKG1* mRNA which has an unusually long (1.5kb) and uridine-rich (28%) 3’ UTR—both relatively rare features in Chlamydomonas mRNAs that tend to have shorter 3’ UTRs (median length 677 bp) and low uridine content (22% ± 3.3% mean and SD) (Methods). We attempted to detect TNY1 binding to *CDKG1* mRNA *in vivo* using RNA crosslinking and immunoprecipitation (RIP) [23] but were unable to amplify an enriched signal due to high background. Instead, we developed an *in vitro* assay where radiolabeled *CDKG1* mRNA fragments were used as a probe for binding to GST-TNY1 fusion protein or GST immobilized on a membrane (Methods) [24]. Radiolabeled *CDKG1* mRNA was synthesized in two fragments, with the 5’ region including the 5’UTR and CDS in one fragment, and the 3’ UTR in a second fragment. After incubation of radiolabeled RNA with membrane-bound GST1-TNY1 or GST1 and washing, the signal was detected only for the 3’ UTR fragment binding to GST1-TNY1 (Fig 6E). These data indicated that TNY1 protein can bind RNA with sequence specificity, including sequences in the 3’ UTR of its likely target gene *CDKG1*.

## Discussion

In this study we identified a new Chlamydomonas sizer protein, TNY1, a hnRNP-related cytosolic RNA binding protein which functions as a negative regulator of cell size in a dosage-dependent manner. Like other size mutants in Chlamydomonas, *tny1-1* mutant cells retain relatively normal cell cycle progression kinetics but do so with altered cell size checkpoints for Commitment and for division number during S/M. As in other systems, size control in Chlamydomonas has at least two components—a size setpoint which governs the optimal target size of daughters measured as the median or modal size of their distribution, and a noise or variance component which describes how accurately cells adhere to a theoretical two-fold size window as the best achievable accuracy for producing daughters by multiple fission [10,11]—and these components are not necessarily the same. In Arabidopsis, budding yeast and mammalian cells, subscaling inhibitor proteins KRP4, WHI5 and RB, respectively, control the size threshold of the G1➔S phase transition and consequently the amount of size variance at this transition [4,5,7,8,25] (S10 Fig). For example, when the Arabidopsis KRP4 subscaling mechanism is genetically disabled, the variance in cell size at G1➔S is not reduced the same as in wild type. At the same time, absolute levels of KRP4 also govern the size setpoint for G1➔S [4]. In contrast, *tny1-1* mutants had altered size setpoints governing Commitment and mitotic size control, but *tny1-1* daughters had similar distribution variance as wild type (S2 Fig) meaning that mechanisms which control variance or noisiness may still operate in *tny1-1* mutants. To date, only *mat3/rbr* mutants seem to have increased noise in daughter size distributions caused by unregulated activity of the cell cycle activator E2F1/DP1 [11] (S1 Table and S2A Fig). However, a definitive analysis of how TNY1 subscaling might influence stochastic processes during cell division will require more in-depth analysis of single cells.

*TNY1* mRNA and TNY1 protein are synthesized once per cell cycle during S/M phase, and TNY1 protein is at its highest concentration in newborn daughters (Fig 4). During G1 phase TNY1 absolute abundance remains constant, meaning that its cellular concentration drops as cells grow. This subscaling behavior appears to be important for size homeostasis since increased or decreased TNY1 dosage or expression impacted mitotic size control (Fig 5). The cell cycle activator and size regulator CDKG1, a D-cyclin dependent RBR kinase is a likely direct target of TNY1 repression since ectopic accumulation of CDKG1 protein and mRNA was observed in *tny1-1* mutants, and TNY1 protein could interact specifically with the 3’UTR of the *CDKG1* mRNA, possibly as a translational repressor or destabilizing factor (Fig 6).

**Fig 7:**
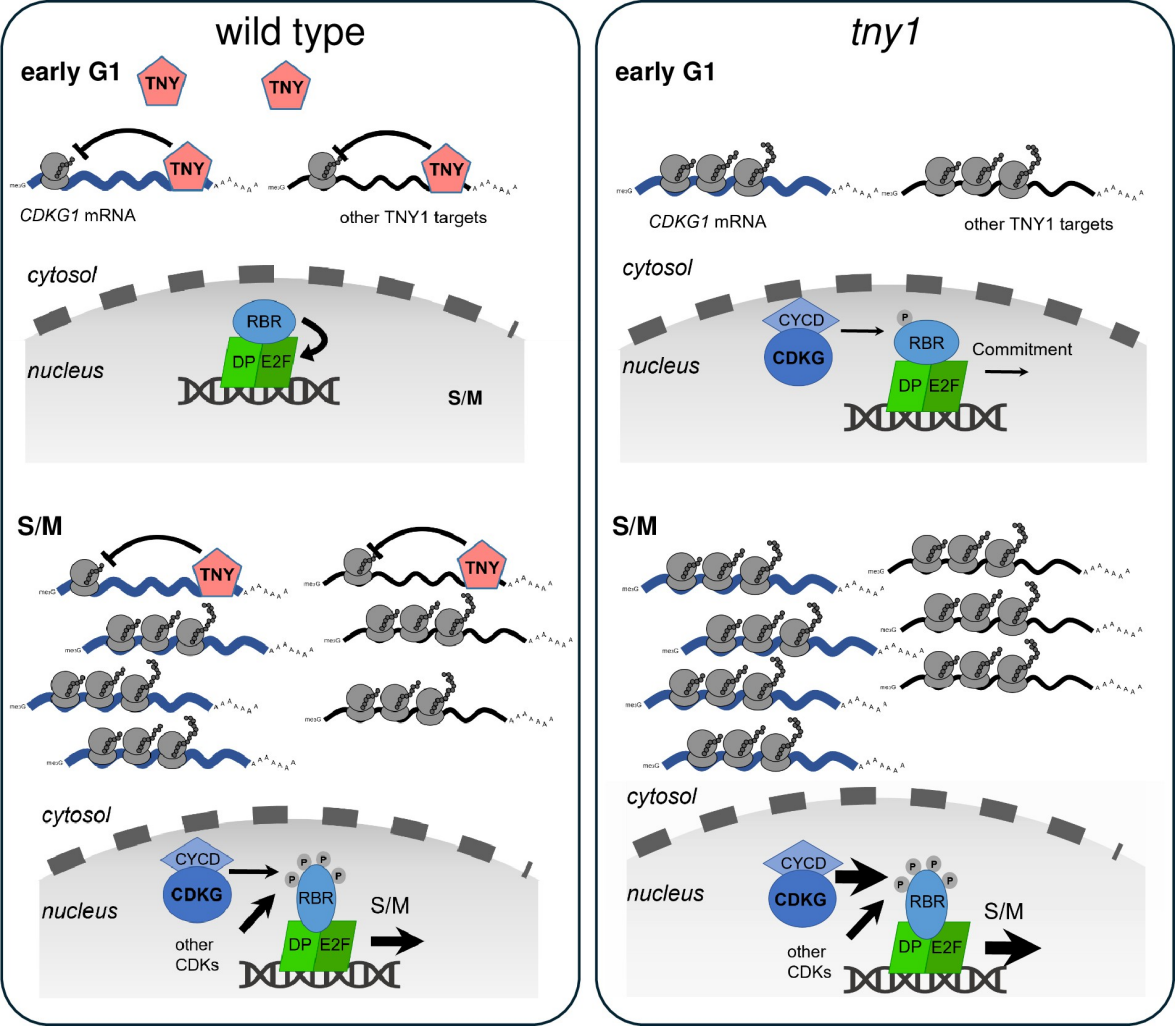
Model for subscaling TNY1 as a regulator of size-dependent cell cycle progression. Left panel, in wild-type cells during early G1 phase (top half) cytosolic TNY1 binds the 3’UTR of *CDKG1* mRNA and possibly other targets and prevents premature expression. Prior to and during early S/M phase (bottom half) *CDKG1* mRNA and other target mRNAs outnumber TNY1 protein which is at its lowest concentration. Translation of CDKG1 drives size-dependent cell cycle progression through phosphorylation of RBR by CDKG1/D-type cyclins and other mitotic kinases in the nucleus [15]. Right panel, in *tny1* mutants some CDKG1 is inappropriately produced in early G1 phase (top half) and may prematurely push cells to Commitment at a smaller size through ectopic phosphorylation of RBR. During S/M phase (bottom half) the absence of TNY1 allows extra CDKG1 to accumulate causing an imbalance in size sensing and more cell divisions than in equivalent-sized wild-type mother cells.

Together these data suggest a model where TNY1 controls cell division by modulating the accumulation of a limiting activator protein, CDKG1, and possibly other limiting cell cycle regulators (Fig 7). This modulation might occur in at least two ways. During G1 phase, CDKG1 is not detectable and does not seem to play a normal role in cells passing Commitment [15], but in a *tny1-1* mutant its inappropriate expression in G1 phase could change the Commitment threshold size by contributing to the premature inactivation of the RBC which controls Commitment cell size [11,13,14]. Just prior to S/M phase, the absence of TNY1 may cause the production of extra CDKG1 leading to increased division number during S/M, or it may cause extra divisions by preventing the timely removal of CDKG1 which normally accompanies mitotic exit (Fig 7). Future experiments based on quantitative detection of CDKG1 in single cells should help resolve whether its abundance is increased in mitotic cells of *tny1-1* cells or whether the postmitotic mis-expression of CDKG1 in *tny1-1* mutants described here (Fig 6) is enough to cause extra cell divisions. *In vivo* binding studies to determine the timing of when TNY1 associates with *CDGK1* mRNA, and to identify other direct RNA targets of TNY1 will be useful for testing the direct repression model for cell size control.

Evidence for cell size checkpoints based on some form of protein subscaling has been found in different eukaryotic taxa, including fungi, animal cells and plant meristems (S10 Fig) [4,5,7,26]. An appealing property of subscaling proteins is their absolute abundance can act as a ruler for perceiving changes in cell size by titrating against an antagonist that remains at constant concentration as cells grow. In the examples cited above (S10 Fig), subscaling is directly tied to DNA or chromatin [3,6]. In budding yeast, Whi5 protein binds to and inhibits the DNA bound transcription factor SBF, a key activator of S phase transcription. While some regulation of Whi5 abundance may occur based on synthesis of Whi5, it is also limited by chromatin binding [5,26,27]. Similar findings were made for the RB protein in mammalian cells which is a functional analog of Whi5 for S phase transcription [7]. In plants, chromatin binding by the CDK inhibitor KRP4 coupled with elimination of excess unbound KRP4 allows daughter cells to be apportioned with a fixed amount of KRP4 that acts as a concentration dependent inhibitor of the cell cycle in the subsequent G1 phase and ensures that S phase entry occurs at a constant average cell size regardless of daughter cell sizes [4]. Here we found that subscaling can also occur for a cytosolic protein, TNY1, that has no direct connection to the nucleus or chromatin. This finding raises the question of how TNY1 synthesis is controlled and how its levels can be modulated so that daughters always contain the same amount of TNY1. One way to achieve a fixed dose of TNY1 per cell would be if production of *TNY1* mRNA is limited by *TNY1* gene copy number in daughters and not influenced by cell size related factors (e.g. transcription factor abundance, co-activator abundance) [28], but this remains to be determined. Supporting this idea, TNY1 absolute abundance in daughters was not influenced by cell size mutants that caused production of large or small daughters (Fig 5A). To date, TNY1 is the only cell cycle regulatory protein in Chlamydomonas known to subscale. The RB complex is downstream of TNY1 in Chlamydomonas, but MAT3/RBR increases in abundance during G1 phase [14,15] and does not show dosage sensitivity for size control as its mammalian homolog RB and its yeast counterpart Whi5 do [5,7]. Thus, the systems-level target for subscaling of size control is not conserved across taxonomic groups.

Interestingly, TNY1 shares some similarity to budding yeast Whi3, an RNA binding protein and negative cell cycle regulator that functions in part by restricting expression of the limiting G1 cyclin Cln3 [29,30]. In budding yeast, Whi3 represses the function of Cdc28-Cln3 by retaining Cdc28-Cln3 complexes in the cytoplasm in G1 phase [31]. Whi3 does not impact the abundance of Cdc28 but does represses *CLN3* mRNA abundance and translational efficiency [32]. In Chlamydomonas, TNY1 functions upstream of CDKG1 and appears to repress the accumulation of *CDKG1* mRNA and CDKG1 protein (Fig 6A–6C). Unlike Whi3, cytosolic TNY1 does not impact the nuclear localization of CDKG1. Musashi proteins (MSIs) are metazoan hnRNPs that play a role in stem cell maintenance and proliferation [33]. While the targets of MSIs are not fully defined, they primarily bind to 3’ UTRs of mRNAs and regulate mRNA stability and/or translation [33,34]. Future work aimed at systems-level understanding of cell size regulatory networks may reveal additional parallels for RNA binding proteins such as TNY1 in governing cell size and cell cycle progression.

## Methods

### Chlamydomonas strains and growth conditions

Strains were maintained on Tris-acetate-phosphate (TAP) + 1.5% agar plates (https://www.chlamycollection.org/methods/media-recipes/tap-and-tris-minimal/). For synchronous growth, strains were cultured at 25°C in Sueoka’s High-Salt-Media (HSM) liquid media [35] with diurnal cycles as indicated and 300 μE total LED light intensity (150 μE blue at 465 nm and 150 μE red at 625 nm) bubbling with 1% CO_2_ in air. Diurnal light regimes used are described in figure legends and text.

Gamete generation, mating, and zygote germination were performed following standard protocols [36–38]. Segregation analysis was done with randomly selected progeny from mating. Dark-shift experiments, Commitment assays, and size distribution measurements with a Coulter Counter (Beckman Multisizer 3) were conducted as described previously [39]. Cell size distribution statistics (mean, median, mode) were determined in data ranging from 20 μm^3^ to 2000 μm^3^. Particle sizes above and below this range are rare, and mostly consist of small debris or large clumps.

### Chlamydomonas transformation

Cells were cultured asynchronously at 25°C in TAP liquid media with constant light 100 uE total light intensity (50% Blue:50% Red– 50 μE blue at 465 nm and 50 μE red at 625 nm LED lights) bubbling with filtered air [40]. Cells were transformed using electroporation as previously described [21]. Transformants were plated on TAP agar plates with either 15 μg/mL of paromomycin or 25 μg/mL of hygromycin depending on selection markers.

### Forward genetic screen for size mutant and mapping of *tny1-1*

Wild type strain CC-124 was subject to an insertional mutagenesis using vector pSI103 [22] linearized with NotI and transformed using the glass bead method [41] with selection on TAP agar plates containing 15 μg/mL paromomycin. Transformants were picked and re-grown in individual wells of 96 well plates, stamped onto TAP agar plates using a 48-pin replicator tool and grown on a light shelf at 25°C for 6 days. Approximately 1/3 of each stamped spot was removed with a toothpick and resuspended in nitrogen-free HSM in a new 96 well plate to create a gamete suspension. Gametes made by growing cells on agar plates for a week provide a less labor-intensive estimate of early G1 phase cells compared with liquid cultures under synchronous growth conditions described above. Gametes were then checked for cell size using a Coulter Counter. Confirmed mutants were then crossed to wild type strain CC-125, and progeny were tested for linkage of the suppressor phenotype to the pSI103 insertion. The *tny1-1* insertion site was determined by sequencing junction fragments from ligation mediated PCR [42]. The insertion site was confirmed using genotyping oligos for *TNY1* and *tny1-1* (S2 Table).

### Diploid generation

Diploid selection was done by plating crosses (described below) shortly after mating on double selection plates to select for both parental markers. Wild type *TNY1/TNY1* vegetative diploids were generated by a mating between wild type CC-1039 (Sager’s 21 gr) (*NIT1 NIT2 MT+*) and wild type CC-124 transformed with pKS-aph7”-lox [43] (*MT-*, hygromycin resistant, *nit1 nit2*) with selection on 25 μg/mL hygromycin and nitrate as the only nitrogen source. Heterozygous *TNY1/tny1* vegetative diploids were generated by a mating between wild type CC-1039 and *tny1-1* (*MT-*, paromomycin resistant) with selection on paromomycin with nitrate as the only nitrogen source. Homozygous *tny1/tny1* vegetative diploids were generated by mating between a *tny1 MT+* Nit+ segregant from a cross with CC-1039 and *tny1* transformed with pKS-aph7”-lox [43], with selection on plates with 25 μg/mL hygromycin and nitrate as the only nitrogen source. Diploid candidates validated by genotyping with mating-type locus oligos (S2 Table) [44].

### Rescue of *tny1-1*

A 3.4 kb fragment containing the full-length genomic region of *TNY1* was amplified from genomic DNA using primers TNY KpnI/TNY NdeI listed in S2 Table. The amplified fragment was digested with KpnI/NdeI and ligated into KpnI/NdeI digested vector pHyg3 (https://www.chlamycollection.org/product/phyg3/) to generate *tny1* rescue construct *pTNY1*. A triple hemagglutinin epitope tag (3xHA) was inserted into *pTNY1* just before the stop codon into a BgllI site created by overlapping PCR with two fragments amplified with oligos TNYKpnI/ TNYBglIIRev and TNY BglIIF/TnyNdeIIF (S2 Table) with Phusion polymerase and GC buffer. A triple HA epitope tag (3xHA) was amplified from 3xHA-MAT3 [14] with oligos HABglII-F/HABglIIR cut with BglII and inserted at the BglII site just before the translation stop codon to generate *pTNY1-3xHA*. *pTNY1* or *pTNY1-3xHA* were transformed into *tny1* by electroporation as described above with selection on TAP agar with 30 μg/mL hygromycin. Individual transformants were picked into 96 well plates and screened for gamete cell sizes as described above for screening insertional mutants. Rescue of the TNY1 protein was confirmed by immunoblotting (see below).

### Mis-expression of *TNY1*

To generate mis-expression construct *pRPL23-TNY1*, full genomic *TNY1* fragment between the start and stop codons was amplified with primers BamHI TNY1 F and Xho1 TNY1 R (S2 Table) with Phusion polymerase and GC buffer from *tny1* rescue construct *pTNY1*. The amplified *TNY1* fragment was digested with BamH1 and Xho1 and inserted into *pRPL23*:*Luc*:*RPL23* [21], then recombined with plasmid pKS-aph7”-lox [43] to generate *pRPL23-TNY1-aph7*. *pRPL23-TNY1-aph7* or *pKS-aph7”-lox* (negative control) were transformed into *tny1-1* by electroporation (see above). Transformants were selected on TAP agar plates containing 25 μg/mL hygromycin.

### Phylogenetic analysis of TNY1 and hnRNP proteins

BLAST searching was done within NCBI or on Phytozome [16] using Chlamydomonas TNY1 protein sequence as a query to find high-scoring hits in plants, green algae and holozoans. Tandem RNA binding domain proteins are found in most eukaryotes, with several representatives besides TNY1 within Chlamydomonas. However, the top BLAST hits for TNY1 were found outside of Chlamydomonas as single best hits within other species of green algae, including three representative volvocine algal species (*Gonium pectorale*, *Tetrabaena socialis*, *Volvox carteri*). The sequences were aligned using MAFFT within Guidance2 [45], and the well-supported portion of the alignment of 158 residues containing the RNA binding domains was retained for phylogenetic analysis. Some duplicates and very closely related sequences were removed to reduce redundancy, with a final group of 39 proteins used for phylogenetic reconstruction. Evolutionary models were tested using Modeltest-NG [46], with the best model being LG+G(1.46)+I(0.08). A maximum likelihood phylogeny was estimated using W-IQ-tree [47] with approximate likelihood ratio testing of branch support.

### 3’UTR analysis

Data on 3’ UTR length and nucleotide composition were extracted from the v5.6 genome assembly and gene models available on Phytozome [16]. 3’ UTR sequences from predicted primary transcripts at each protein coding locus were used to determine length distributions and nucleotide composition. The length data were comparable to those from a prior analysis done with an earlier version of the genome assembly and gene models [48].

### TNY1 antibody generation

A full length *TNY1* cDNA was amplified with primers TNY1-1F and TNY1-1R (S2 Table) from cDNA prepared using RNA from wild-type strain CC-124 and inserted into pGEM-T easy vector (Promega) to generate *pGEM-TNY1*. After verification by Sanger sequencing the *TNY1* cDNA fragment was released by digestion with NdeI and XhoI (NEB) and inserted into vector pET28a (Sigma-Aldrich) digested with NdeI and XhoI. The construct was transformed into *E*.*coli* strain BL21 codon plus-RIL (DE3) (Agilent technologies). Induction of recombinant TNY1 expression in *E*. *coli* and purification of insoluble 6xHis-TNY1 was performed under denaturing conditions as described previously [14]. Purified 6xHis-TNY1 was cut out from a Coomassie blue stained SDS-PAGE gel and sent to Cocalico Biological Inc. to generate rabbit polyclonal anti-sera. Polyclonal antibodies were affinity purified with AminoLink Plus Resin (Thermo Fisher) coupled to purified GST-TNY1 (see below).

### Protein extraction

Chlamydomonas cultures were grown as described above and harvested by centrifugation at 4000g for 5 min after adding Tween-20 to a final concentration of 0.005%. Pellets were washed in PBS and resuspended in lysis solution (1xPBS pH 7.4, 1x Sigma plant protease Inhibitor, 5 mM Na_3_VO_4_, 1 mM NaF, 1 mM Benzamidine, 500 mM PMSF, 1 μM ALLN, 1 μM MG-132) to a final concentration of 5x10^8^ cells/mL, and immediately frozen in liquid nitrogen. Pellets were thawed quickly and placed on ice. Resuspensions were then processed with a Covaris ultrasonicator (peak power 150, duty factor 150, cycle 200, treatment 120 sec) to generate protein lysates.

### Immunoblotting

Lysate quantity for loading in each lane was determined from measuring cell number from each sample prior to preparation and protein concentration of the lysate (see below). Between 9 and 18 ug total protein were loaded per lane for equal protein loading, and 5x10^4^ synchronous G1 phase cells (or same volume of culture for mitotic time points) for equal cell number loading. Protein lysates were mixed 5:1 with 6X SDS protein loading buffer and boiled for 10min. Lysates were cleared by centrifugation at 12,000 g for 10 min. Total protein was separated on 12% SDS-PAGE gels and wet-transferred to PVDF membranes at 50 Volt for 1 hr. When TotalStain Q (PVDF) was used, membranes were stained according to manufacturer’s instructions immediately after transfer. After quantitation of total protein using TotalStain Q staining (see below), membranes were blocked in PBS containing 9% nonfat dry milk for 1 hr at RT, then incubated overnight for 16hrs at 4°C with primary antibodies (1:5000 α-TNY1, 1:10,000 Roche α-HA high affinity 3F10, 1:50,000 Sigma-Aldrich α-Tubulin, or 1:50,000 Invitrogen α-Histone H3) in 5% non-fat dry milk. Membranes were then washed in PBS containing 0.1% Tween 4 x 15 min, incubated at room temperature with secondary antibodies coupled to horseradish peroxidase (1:20,000 Thermo Fisher goat-anti-rabbit, or 1:20,000 Millipore Sigma goat-anti-rat in 5% nonfat dry milk). Membranes were washed again in PBS containing 0.1% Tween 4 x 15 min, then subject to chemiluminescent detection using autoradiographic film or a Bio-Rad quantitative imaging system (Chemi Doc XRS+ Imaging System) for quantitative experiments (see below).

### Total protein quantification

Protein input for cell lysate was determined using a Pierce BCA Protein Assay Kit (Thermo Fisher Scientific) with bovine serum albumin as a standard. TotalStain Q (PVDF) (Azure Biosystems) staining was performed for total protein input quantification following the manufacturer’s protocol using a Sapphire FL Biomolecular Imager (Azure Biosystems). Fluorescent signal in a range of 9 to 75 μg of input lysate used for quantitative experiments was close to linear (S6A and S6B Fig). An image with the longest exposure settings yet without pixel saturation was taken. All the lanes were then automatically detected, along with an empty lane on the same blot to represent the background signal. The TotalQ signal in each lane was computed as (total signal-background). All quantitative blots were arbitrarily rescaled with a maximum value of 1. TotalStain Q (PVDF) is compatible with subsequent immunoblotting and chemiluminescence detection.

### Immunoblotting signal quantification

Bio-Rad Image-Lab (PC version) software was used for image capture and processing. “High Sensitivity ChemiBlot” setting was used with accumulated exposure time (300 seconds/100 images) to take a stack of 100 raw images with different exposure times. The image with the longest exposure time but no pixel saturation was chosen for signal measurement. Under “Volume Tool,” “Rectangular” boxes were drawn to outline each band, along with a control region above or below the band to control for background signal. The signal for each lane was the computed as (boxed band signal-background signal). For some experiments histone H3 was used as a control for cell numbers. Tubulin or TotalStain Q were used as loading controls for protein input. Immunoblot signals were relatively linear using α-TNY1, α-Tubulin, and α-Histone H3 antibodies in a range of 2–16 μg total protein per lane (S6C Fig).

### qRT-PCR

Total RNA samples were extracted at different time points from synchronized strains using a Trizol-like reagent following the method of [13] then digested with RNase-free Turbo DNase following the manufacturer’s protocol. 4 μg total RNA was reverse transcribed with oligo dT and random hexamers (9:1) using Thermo Script Reverse Transcriptase at 25°C for 10 min, 42°C for 10 min, 50°C for 20 min, 55°C for 20 min, 60°C for 20 min, 85°C for 5 min. SYBR-Green based qPCR reactions in two technical duplicates of two biological replicates were performed and quantitated in a Bio-Rad CFX384 system. Each 10 μL reaction contained 0.1 μL cDNA, 1x Invitrogen Taq buffer, 3.5 mM MgCl_2_, 0.5x SYBR Green I, 0.05% Tween 20, 0.05 mg/mL BSA, 5% DMSO, 200 μM dNTPs, 0.3 μM primers, and 5U of Invitrogen Taq DNA polymerase. Expression was normalized against *GBLP* (GenBank NC_057009.1) as an internal control. The melting curve was examined for each reaction to ensure that no primer dimers or non-specific PCR products were present. qPCR experiments were performed targeting *CDKG1*, *TNY1*, and *GBLP* (S2 Table).

### Light microscopy

Chlamydomonas cells were fixed in 0.2% glutaraldehyde final concentration. Cells were mounted on slides and imaged with a Leica DMI 6000 B microscope with a 63x oil objective (NA 1.40) and DIC optics with images taken using a Photometrics Coolsnap HQ2 CCD camera.

### Immunofluorescence microscopy

Wild type CC-125, *TNY1-HA*::*tny1*, *HA-gCDKG1*:: *cdkg1-2* [15], or *HA-gCDKG1*:: *cdkg1-2 tny1-1* strains were synchronized as described above on a 14hr light: 10hr dark diurnal cycle. S/M phase cells were collected at ZT 15 hrs and daughter cells at ZT 23 hrs. Cells were centrifuged and collected in an Eppendorf tube, fixed with 2% paraformaldehyde in PBSP (1x PBS pH7.4, 1 mM DTT, 1x Sigma plant protease inhibitor cocktail) for 30 min on ice. Fixed cells were extracted in cold methanol 3 x 10 min at -20°C and rehydrated in PBSP for 30 min on ice. Cells were blocked for 30 min in blocking solution I (5% BSA and 1% cold water fish gelatin in PBSP) and 30 min in blocking solution II (10% goat serum, 90% blocking solution I). Cells were incubated overnight with primary antibody α-HA Roche HA high affinity 3F10 (1:1000 dilution in 20% blocking solution I) at 4°C, then washed 3 x 10 min in 1% blocking solution I at room temperature. Cells were then incubated with 1:1000 Alexa Fluor 568 conjugated goat anti-mouse IgG in 20% blocking solution I for 1 hr at 4°C and then incubated with 4’,6-Diamidino-2-Phenylindole, Dihydrochloride (DAPI) at a final concentration of 5ug/mL for 5 min. Cells were washed in 1 x PBS for 3 x 10 min. Cells were mounted in 9:1 Mowiol: 0.1% 1, 4-phenylenediamine (PPD), and imaged with a Leica DMI 6000 B microscope with a 63x oil objective (NA 1.40) and a Photometrics Coolsnap HQ2 CCD camera. Fluorescence illumination was provided by a metal halide lamp (Prior Lumen 200 Fluorescence Illumination Systems) using a Leica A4 filter cube (ex 360/40; em 470/40) for DAPI imaging and TX2 filter cube (ex 560/40; em 630/75) for detection of HA-TNY1 or HA-CDKG1.

### Confocal Immunofluorescence microscopy

*HA-gCDKG1*:: *cdkg1-2* or *HA-gCDKG1*:: *cdkg1-2 tny1-1* cells were stained and mounted in microscopy slides as described above. Cells were imaged using a Leica SP8-X confocal microscope equipped with a white light laser and a 405 nm diode laser using 63x/1.20 water objective. DAPI DNA staining was detected using a Leica HyD detector with 405 nm excitation and a 440–470 nm emission window. HA-CDKG1 was detected using 578 nm excitation and a and a 580–620 nm emission window. Frame average = 1. Line average = 16. Frame accumulation = 3. Line accumulation = 1. Bright Field images were captured using a PMT trans detector.

### Construction of a TNY1-mCherry expressing strain

To generate a fluorescence protein-tagged *tny1* complemented strain, a *pTNY1*:*gTNY1-GFP-TNY1 3’ UTR* construct was generated first. Chlamydomonas codon optimized GFP fragment (SpeI-SacI-BamHI-GFP-Xba-Xho-EcoR-NcoI) was amplified from *pMF124cGFP* [49] and digested by SpeI and NcoI, followed by insertion into *RPL23*:*Luc*:*RPL23* which is digested by XbaI and NcoI. A fragment of *pTNY1*:*gTNY1*, including the promoter region, 5’UTR, and exons and intron of genomic TNY1, was amplified and digested with SacI and BamHI, and inserted into the above modified GFP plasmid. TNY1 3’UTR and terminator region was amplified and digested with XbaI and EcoRI, followed by insertion into the above *pTNY1*:*gTNY1-GFP* backbone. Chlamydomonas codon-optimized mCherry was amplified using a primer set of BamH1 mCherry F and XbaI mCherry R (S2 Table) from pLM006 [50], digested with BamH1 and Xba1, then used to replace GFP in the plasmid *pTNY-GFP* digested with BamHI and XbaI to create plasmid *pTNY1-mCherry*. *pTNY1-mCherry* was transformed into *tny1-1* and rescued transformants were identified by measuring gamete sizes as described above and then confirmed by immunoblotting with α-TNY1 and measuring sizes of daughter cells.

### Confocal live cell fluorescence microscopy

*pTNY1-mCherry* expressing transformants were synchronized and harvested throughout the cell cycle. Live cells were immobilized on a very thin layer of TAP agar on a glass slide, and topped with a coverslip, which was sealed with PicoDent following the manufacturer’s instructions (https://www.picodent.de/). Cells were imaged using a Leica SP8-X confocal microscope equipped with a white light laser and a 405 nm diode laser using 63x/1.20 water objective. TNY1-mCherry was detected using a Leica HyD detector with 570 nm excitation and a 550–650 nm emission window. Frame average = 1. Line average = 16. Frame accumulation = 4. Line accumulation = 1. Fluorescence lifetime gating 0–4.9 ns was used to remove most of the chlorophyll background/bleed-through signals. Chlorophyll was detected using 405 nm excitation and a 676–704 nm emission window. Bright Field images were captured with a PMT trans detector.

### Native gel separation and detection of TNY1 RNP complexes

50 mL samples from Chlamydomonas cultures at 10^6^ cells/mL were mixed with Tween-20 to a final concentration of 0.005% and collected by centrifugation at 4000 g for 5 min. Pellets were washed in PBS and resuspended in lysis solution (1xPBS pH 7.4, 1x Roche plant protease Inhibitor, 1 mM PMSF) to a final concentration of 5x10^8^ cells/mL, and immediately frozen in liquid nitrogen. Pellets were thawed on ice and centrifuged at 12,000 g for 10 min at 4°C.

For RNA binding assays, 20 μL of supernatant was incubated with different RNase dilutions 1:10, 1:100 or 1:1000 (stock 10 mg/mL, NEB) or with 1:10 DNase I (stock 2 U/μL, Roche), and micrococcal nuclease (stock 2000 U/μL, NEB). 6 X SDS protein loading buffer without DTT nor SDS was added to samples before loading into a precast native 4–12% tris glycine gel (Invitrogen) without SDS in Tris-Glycine running buffer. A mixture containing aldolase, BSA and ferritin was used as a molecular weight marker. Native PAGE gels were transferred to nitrocellulose membranes in 25 mM Tris, 192 mM glycine, 20% methanol. Blots were blocked in 1x PBS with 5% non-fat dry milk for 1h at room temperature and incubated with 1:2500 anti-TNY diluted in PBST (PBS + 0.05% Tween-20) with 3% dry milk at 4°C overnight. After washing in PBST for 3* 10 min, the blot was incubated with horseradish peroxidase (HRP) conjugated goat-anti-rabbit-IgG (1:5000, Pierce ECL) for 1hr at RT, then washed in PBST for 3* 10 min, and processed for chemi-luminescence (Luminata forte, Millipore).

### ^32^P RNA radio-labeling

*CDKG1* DNA for *in vitro* transcription was amplified from genomic DNA with oligos containing a T7 promoter (S2 Table). ^32^P labeled RNA was generated/transcribed *in vitro* using a Maxiscript kit in the presence of α-^32^P-CTP (NEN Radiochemicals) according to manufacturer instructions. Each 25 μL reaction had the following components: DNA template 0.5ug, 10x Transcription buffer 2 μL, 0.5 mM ATP, 10mM GTP 1 μL, 10mM UTP 1 μL, 500uM CTP 1 μL, ^32^P-CTP 2 μL (10 mCi/mL), 2 μL T7 RNA polymerase. After 1 hr reaction at 30°C, the mixture was treated with DNaseI (ambion) and purified with Sigma post reaction clean-up columns SigmaSpin to remove unincorporated nucleotides. RNA integrity was visualized by separating a sample of the RNA on a urea denaturing 4% polyacrylamide gel followed by autoradiography.

### GST-TNY recombinant protein expression

The *TNY1* cDNA coding sequences were cloned into the Gateway pDEST15-GST (glutathione S-transferase) plasmid using the procedures recommended by the manufacturer (Invitrogen) with oligos listed in S2 Table. GST-TNY constructs were transformed into *E*.*coli* BL21 codon plus-RIL strain (Agilent Technologies). Cells were grown in LB media and induced for 5 hrs at 30°C with 0.5 mM isopropyl-β-d-thiogalactopyranoside (IPTG) when cultures reached an O.D.600 of 0.5. After induction, cells were harvested by centrifugation and dry cell pellets stored at -80°C. Frozen cells were thawed on ice and resuspended in 1/10^th^ original culture volume of EB (100 mM Tris-HCl, pH 8.0, 500 mM NaCl, and 10 mM imidazole), sonicated eight times for 2 min each on ice with a Branson sonicator (50% power with a duty cycle of 0.5s on and 0.5 s off) followed by supernatant clearance by centrifugation at 12,000g for 10 min. GST-TNY recombinant proteins were purified from the soluble fraction using Glutathione Sepharose beads (Amersham) following the product manual.

### TNY1 RNA binding assay

Equal amounts of GST purified proteins estimated based on Ponceau S staining were separated by SDS-10% PAGE and transferred to a nitrocellulose membrane (0.22-m pore size) and stained with homemade Ponceau S. The membrane was incubated at 4°C overnight with renaturation buffer: 50 mM tris-HCl pH 7.5, 100 mM KCl, 1% Triton X-100 and 10% glycerol. After renaturation, the membrane was incubated for 1 hr with reactivation buffer (Tris-HCl pH 7.5, 0.1% triton X-100, 10% glycerol) at room temperature, blocked for one hour with yeast tRNA (80 μg/mL) in reactivation buffer followed by incubation with ^32^P labeled RNA in reactivation buffer for 3 hrs. Membranes were washed 4X with reactivation buffer and exposed to X-ray film for 2 days at -80°C before development.

## Supporting information

S1 Fig Characterization of *tny1-1* and rescued *tny1-1* strains. (A) Plot showing passage through Commitment (Commitment %, solid lines) and mitotic index (fraction dividing %, dashed lines) of synchronous *tny1-1*, wild type CC-124, and a *tny1-1* rescued strain *gTNY1*::*tny1-1* collected at indicated time points during a synchronous diurnal cycle. Grey dotted line marks the time when 50% of the cells had passed Commitment (~ZT 6 hrs). (B) Plot of modal cell sizes for cultures in panel (A). Grey dotted line marks at ZT 6hrs, ~50% of the cells had passed Commitment in all the genotypes. Commitment sizes for each genotype: *tny1 ~* 80 μm^3^, wild type and *gTNY1*::*tny1-1 ~* 200 μm^3^. (C) Division number profiles of *tny1-1* and wild type CC-124. Cells from synchronized cultures were collected at indicated times, plated on minimal media, incubated in the dark, and scored for cell division number (see Methods). ~100 clusters were scored for each genotype at each time point. Two independent repeats were plotted side by side (rep1 and rep2). (D) Division number profiles of size-matched G1 phase cultures of *tny1-1* and wild type cells (~230 μm^3^) taken at different time points in G1 to enable *tny1-1* cultures to reach the same size as wild type. A summary of the results is presented in the table.
(TIF)

S2 Fig Characterization of *tny1-1* and rescued *tny1-1* strains (continued-1). (A) Statistics on log2 transformed size histogram data for synchronous daughter cells (ZT 0 equivalent) of size mutants and wild type. (B) Statistics on log2 transformed size histogram data for synchronous *tny1-1* and wild type CC-124 in G1 phase at different ZT hrs.
(TIF)

S3 Fig Characterization of *tny1-1* and rescued *tny1-1* strains (continued-2). (A) Plot showing timing of Commitment for indicated genotypes similar to panel S1A Fig. Grey dotted lines mark Commitment timing of *cdkg1-2* or *tny1-1 cdkg1-2* and wild type. (B) Plot of modal cell sizes for cultures in panel S3A Fig. Grey dotted lines mark cell sizes of strains showing that *cdkg1-2* and *tny1-1 cdkg1-2* have similar Commitment sizes as wild type. *cdkg1-2* and *tny1-1 cdkg1-2* pass Commitment at an earlier ZT. Commitment sizes for each genotype: *tny1 ~* 80 μm^3^; wild type, *gTNY1*::*tny1-1*, *tny1-1 cdkg1-2*, and *cdkg1-2 ~* 200 μm^3^. (C) Linkage between paromomycin insertion (Fig 1A) and small size phenotype. Each data point represents the modal size of a population derived from an independent meiotic progeny of *tny1-1* crossed to wild-type strain CC125 and grouped according to their paroR (*tny1-1* insertion) or paroS (*TNY1*) phenotypes. Box and whisker plots of modal gamete sizes for paroS (n = 44) or paroR (n = 46) progeny. Boxes enclose the second quartile of data with horizontal lines showing median values, and whiskers enclose the 10^th^ - 90^th^ percentiles. Outliers are plotted as individual data points. The size distributions were significantly different in a Student’s t-test (*, p<0.01). (D) Validation of genotyping primers for *tny1-1*, *TNY1*, and mating type loci (mating type *minus*, *mt-*; mating type *plus*, *mt+*) (see S2 Table). (E) Growth on selective media for *tny1-1* (paromomycin resistance marker; Paro) and *tny1-1* with rescuing constructs (with hygromycin resistance markers, Hyg).
(TIF)

S4 Fig Multiple sequence alignment of green algal TNY1 orthologs. Peptide alignments for subset of proteins from Fig 2: *Chlamydomonas reinhardtii* TNY1 (Cre07.g330300), *Volvox carteri* (Vocar.0031s0001), *Chromochloris zofingiensis* (Cz12g11070), and *Dunaliella salina* (Dusal.0065s00006). Gene IDs are from Phytozome [16]. Alignment is shaded to show conserved residues. Positions of RNA recognition motifs 1 and 2 (RRM1, RRM2) and a conserved C-terminal motif (CM) are marked. The inverted black triangle shows the position of the single intron found in TNY1 orthologs in the green algal subclade.
(TIF)

S5 Fig Detection of TNY1-mCherry expression in *gTNY-mCherry*:*tny1-1* strains. (A) Immunoblots with whole cell lysates of daughter cells from indicated genotypes. The gel was loaded with equal protein per lane, fractionated by SDS PAGE, and immunoblotted using α-TNY1 (upper panel). Coomassie blue (CBB) staining is shown in the lower panel as a loading control. (B) Size distributions of daughter cells from *tny1-1* (median size 55 μm^3^/modal size 46 μm^3^), a *tny1* rescue strain *gTNY1*:*tny1-1* (median size 79 μm^3^/modal size 70 μm^3^), and two independent mCherry tagged rescue *TNY1-mCherry*::*tny1* strains (strain c2.2 median size 81 μm^3^/modal size 83 μm^3^, strain c2.6 median size 86 μm^3^/modal size 79 μm^3^). Median sizes of *TNY1-mCherry*::*tny1* transformants and *gTNY1*:*tny1-1* rescued strains are not different (p>0.1, Student’s t-test) (S1 Table). (C) Fields of *TNY1-mCherry*::*tny1* cells, along with *gTNY1*::*tny1* cells as the negative control under the same detection settings. Annotation is the same as Fig 3. Scale bar = 20 μm. (D) DIC and immunofluorescence microscopy images of wild type CC-124 and *gTNY1-HA*::*tny1-1*. Daughter cells were fixed and immunostained for HA epitope (pseudo-colored green). DNA was stained with DAPI (pseudo-colored red). Merged fluorescence images (Overlay). Scale bar = 10 μm.
(TIF)

S6 Fig Cell cycle and diurnal control of *TNY1* mRNA and TNY1 protein accumulation. (A) and (B) Protein lysates were made using indicated cell numbers of wild-type daughter cells. Protein quantity was determined using a standard BCA kit with two replicates for each standard concentration. TotalStain Q staining signal across a range of loading amounts are documented with two replicates per sample. The band of the highest signal was set to be 1 in each blot. (A) Protein loading range used for most experiments with 9–30 μg/lane. (B) Expanded total protein loading dilution series with 9–75 μg/lane. The grayscale images were of the longest exposures without any saturated pixels. Linear regression lines (grey) are plotted for each data series. (C) Immunoblots with protein lysates made using indicated cell numbers of wild type daughter cells. Over the normal protein loading range the signals of α-TNY1, α-Histone H3, α-Tubulin are approximately linear. The band with the highest signal was set to 1 in each plot. Two independent replicates were plotted side by side (rep1 and rep2) with linear regression plotted from the average of the two repeats (black dots) for each antibody. (D) Representative size distributions of a synchronous wild type strain CC-124 at different ZT time points throughout a standard 12hr:12hr light:dark cycle. Protein lysates at each ZT were collected for immunoblots.
(TIF)

S7 Fig Cell cycle and diurnal control of *TNY1* mRNA and TNY1 protein accumulation (continued). (A)–(E) Immunoblot repeats as described in Fig 4B. Three biological replicate sets with two technical repeats are included as follows: biological replicate set 1—S7A and S7B Fig; biological replicate set 2—Figs 4B and S7C; biological replicate set 3—S7D and S7E Fig. (F) Data were plotted as in Fig 4C with the inclusion of total protein (grey bars) from TotalStain Q staining with the ZT1 value set to 1. Bar values/dots represent the average of three biological repeat sets with two technical replicates each. Error bars: standard deviation of three biological replicates. (G) Size distributions of mitotic populations at ZT 15 under different diurnal regimes in Fig 4A. Standard regime at ZT 15, mean cell size 511 μm^3^. Early dark regime at ZT 15, mean cell size 341 μm^3^. Extended light regime at ZT 15, mean cell size 581 μm^3^.
(TIF)

S8 Fig Dosage sensitivity of *TNY1*. (A) Daughter size distributions (ZT 0 equivalent) of dark-shifted size mutants compared with a wild-type strain. *mat3-4/rbr* (median size 37 μm^3^/modal size 28 μm^3^), wild type (median size 73 μm^3^/modal size 75 μm^3^), *dp1-1* (median size 111 μm^3^/modal size 123 μm^3^), and *cdkg1-2* (median size 114 μm^3^/modal size 108 μm^3^). (B) Immunoblot of samples in Fig 5A with gel loading by equal protein per lane with signal quantitation shown in bar plots below. Annotation is the same as Fig 5A. (C) Left panel, box and whiskers plots of modal gamete sizes of populations derived from a back-cross between wild type CC124 and rescued strains g*TNY*::*tny1-1* (left side) or *gTNY*::*tny1-1* (right side). Each data point represents the modal size of a gamete population derived from an independent meiotic progeny. Numbers of progeny for each genotype sampled are listed in the table above each plot. Boxes enclose the second quartile of data with horizontal lines showing median values, and whiskers enclose the 10^th^ - 90^th^ percentiles. Outliers are plotted as individual data points. Comparisons among the four genotypes were done using a one-way ANOVA with post-hoc Tukey HSD Test. *, samples are different at p < 0.01; n.s., samples are not significantly different (p>0.05). (D) Plot showing timing of passing Commitment for indicated genotypes, similar to S1A Fig. Grey dotted lines mark Commitment timing of *tny1-1*, wild type, and a *RPL23*:*TNY tny1-1* strain with a large size phenotype. Plot of modal cell sizes for cultures in panel (D). Grey dotted lines mark cell sizes of strains in panel (E) showing that *tny1-1*, wild type, and the *RPL23*:*TNY tny1-1* strain pass Commitment at about the same ZT. Commitment sizes for each genotype: *tny1 ~* 80 μm^3^, wild type *~* 200 μm^3^, *RPL23*:*TNY tny1-1* #1 ~ 250μm^3^.
(TIF)

S9 Fig TNY1 inhibits the accumulation of CDKG1 protein. (A) Size distributions of synchronous mitotic (ZT 13) and post-mitotic (ZT 1) populations of indicated strains. (B) Immunoblots using synchronized strains of indicated genotypes loaded with equal numbers of cells per lane and probed with α-HA to detect HA-CDKG1 or stained with Coomassie blue (CBB). (C) Immunofluorescence images of *HA-CDKG1*::*cdkg1* and *HA-CDKG1*::*cdkg1 tny1* post-mitotic cells (ZT 1) as described in Fig 6C. Scale bar = 10 μm.
(TIF)

S10 Fig Systems level comparison of cell size control across taxa. Cell cycle inhibitors subscaling with cell size in G1 phase are highlighted in bold red.
(TIF)

S1 Table Size distribution statistics for selected strains used in this study. (XLSX)

S2 Table Oligonucleotides used in this study. (XLSX)

S1 Data Coulter Counter size distribution files for Figs 1B, 1C, 5B, 5D, S1C, S1D, S2A, S2B, S5B, S6D, S7G, S8A, and S9A. (XLSX)

S2 Data Immunoblot quantification for Figs 4B and S7A–S7E. (XLSX)
