# Supplementary figures and images for: Subscaling of a cytosolic RNA binding protein governs cell size homeostasis in the multiple fission alga Chlamydomonas
Source: PLoS Genet. 2024 Mar 18;20(3):e1010503. doi: 10.1371/journal.pgen.1010503 (PMC10977881; doi:10.1371/journal.pgen.1010503)

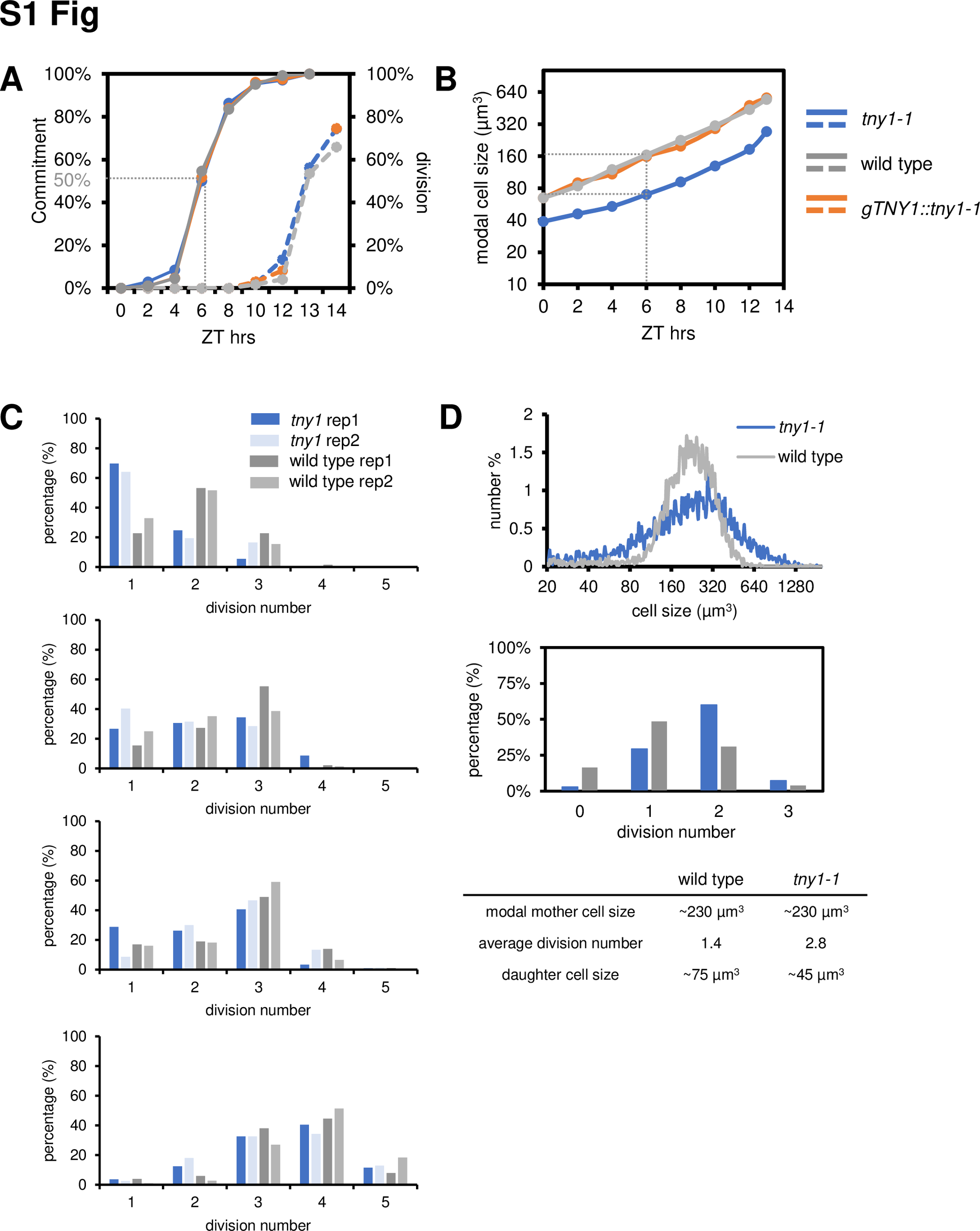

Supplement: S1 Fig — (A) Plot showing passage through Commitment (Commitment %, solid lines) and mitotic index (fraction dividing %, dashed lines) of synchronous tny1-1, wild type CC-124, and a tny1-1 rescued strain gTNY1::tny1-1 collected at indicated time points during a synchronous diurnal cycle. Grey dotted line marks the time when 50% of the cells had passed Commitment (~ZT 6 hrs). (B) Plot of modal cell sizes for cultures in panel (A). Grey dotted line marks at ZT 6hrs, ~50% of the cells had passed Commitment in all the genotypes. Commitment sizes for each genotype: tny1 ~ 80 μm3, wild type and gTNY1::tny1-1 ~ 200 μm3. (C) Division number profiles of tny1-1 and wild type CC-124. Cells from synchronized cultures were collected at indicated times, plated on minimal media, incubated in the dark, and scored for cell division number (see Methods). ~100 clusters were scored for each genotype at each time point. Two independent repeats were plotted side by side (rep1 and rep2). (D) Division number profiles of size-matched G1 phase cultures of tny1-1 and wild type cells (~230 μm3) taken at different time points in G1 to enable tny1-1 cultures to reach the same size as wild type. A summary of the results is presented in the table. (TIF) [file pgen.1010503.s001.tif]

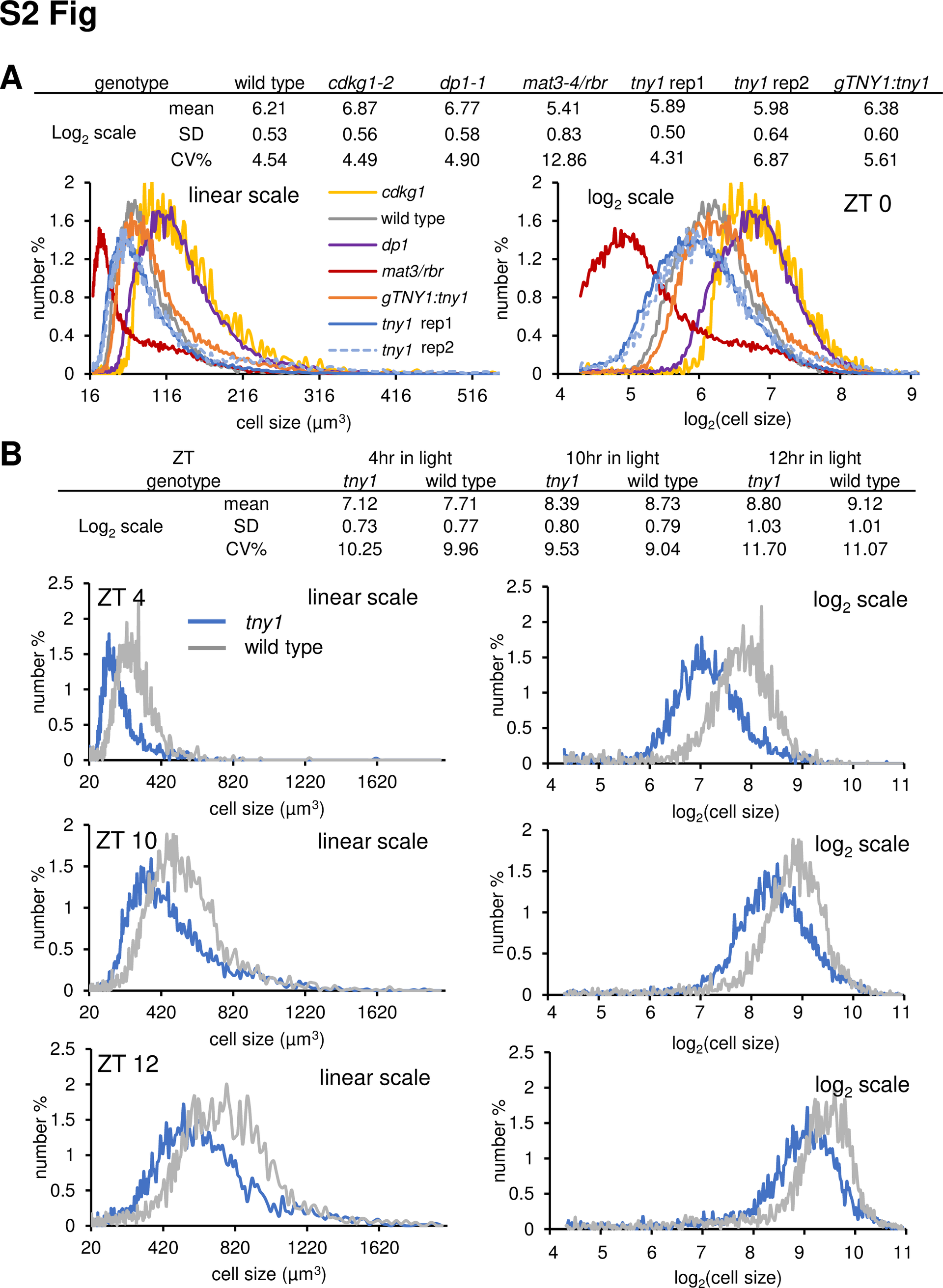

Supplement: S2 Fig — (A) Statistics on log2 transformed size histogram data for synchronous daughter cells (ZT 0 equivalent) of size mutants and wild type. (B) Statistics on log2 transformed size histogram data for synchronous tny1-1 and wild type CC-124 in G1 phase at different ZT hrs. (TIF) [file pgen.1010503.s002.tif]

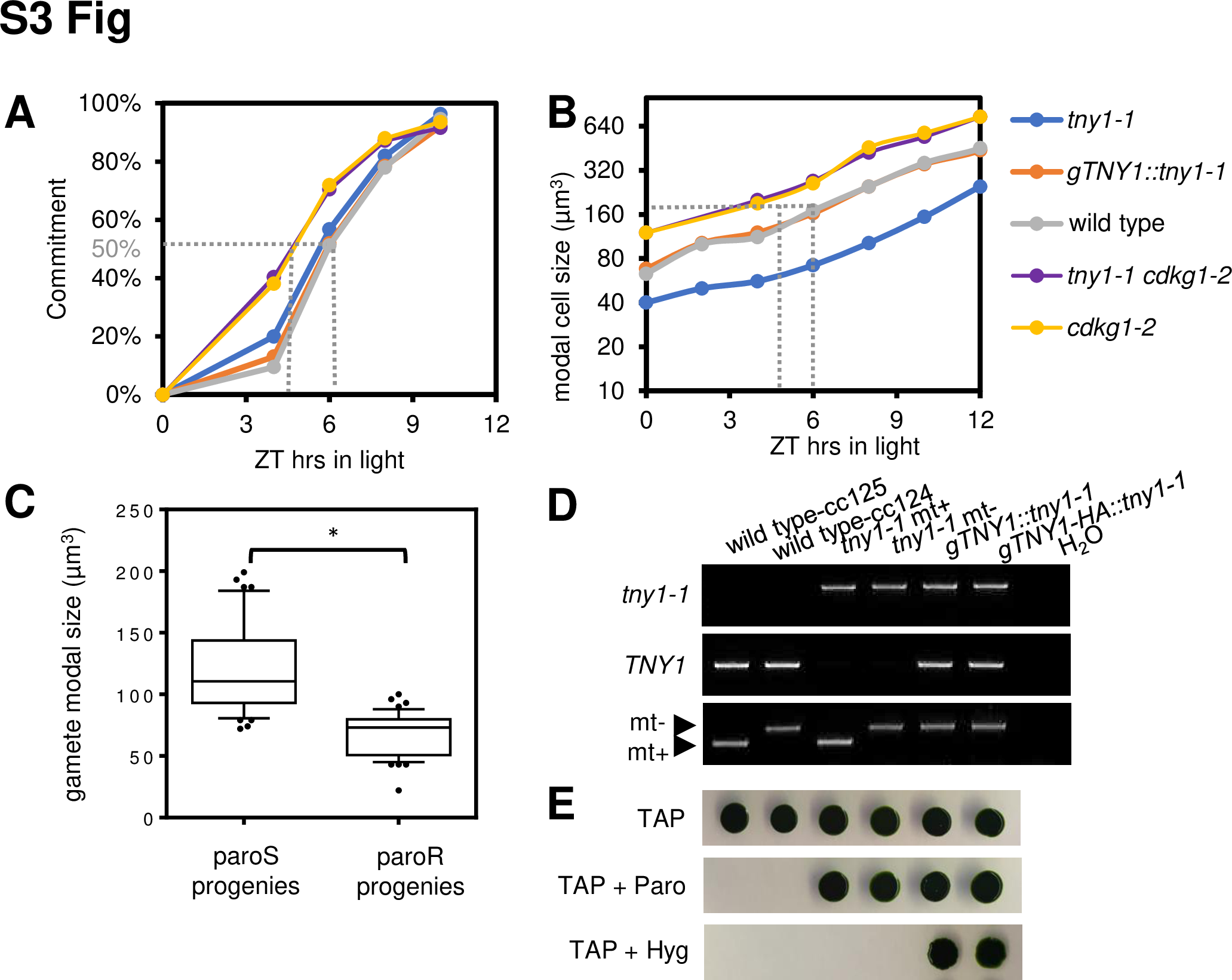

Supplement: S3 Fig — (A) Plot showing timing of Commitment for indicated genotypes similar to panel S1A Fig. Grey dotted lines mark Commitment timing of cdkg1-2 or tny1-1 cdkg1-2 and wild type. (B) Plot of modal cell sizes for cultures in panel S3A Fig. Grey dotted lines mark cell sizes of strains showing that cdkg1-2 and tny1-1 cdkg1-2 have similar Commitment sizes as wild type. cdkg1-2 and tny1-1 cdkg1-2 pass Commitment at an earlier ZT. Commitment sizes for each genotype: tny1 ~ 80 μm3; wild type, gTNY1::tny1-1, tny1-1 cdkg1-2, and cdkg1-2 ~ 200 μm3. (C) Linkage between paromomycin insertion (Fig 1A) and small size phenotype. Each data point represents the modal size of a population derived from an independent meiotic progeny of tny1-1 crossed to wild-type strain CC125 and grouped according to their paroR (tny1-1 insertion) or paroS (TNY1) phenotypes. Box and whisker plots of modal gamete sizes for paroS (n = 44) or paroR (n = 46) progeny. Boxes enclose the second quartile of data with horizontal lines showing median values, and whiskers enclose the 10th - 90th percentiles. Outliers are plotted as individual data points. The size distributions were significantly different in a Student’s t-test (*, p<0.01). (D) Validation of genotyping primers for tny1-1, TNY1, and mating type loci (mating type minus, mt-; mating type plus, mt+) (see S2 Table). (E) Growth on selective media for tny1-1 (paromomycin resistance marker; Paro) and tny1-1 with rescuing constructs (with hygromycin resistance markers, Hyg). (TIF) [file pgen.1010503.s003.tif]

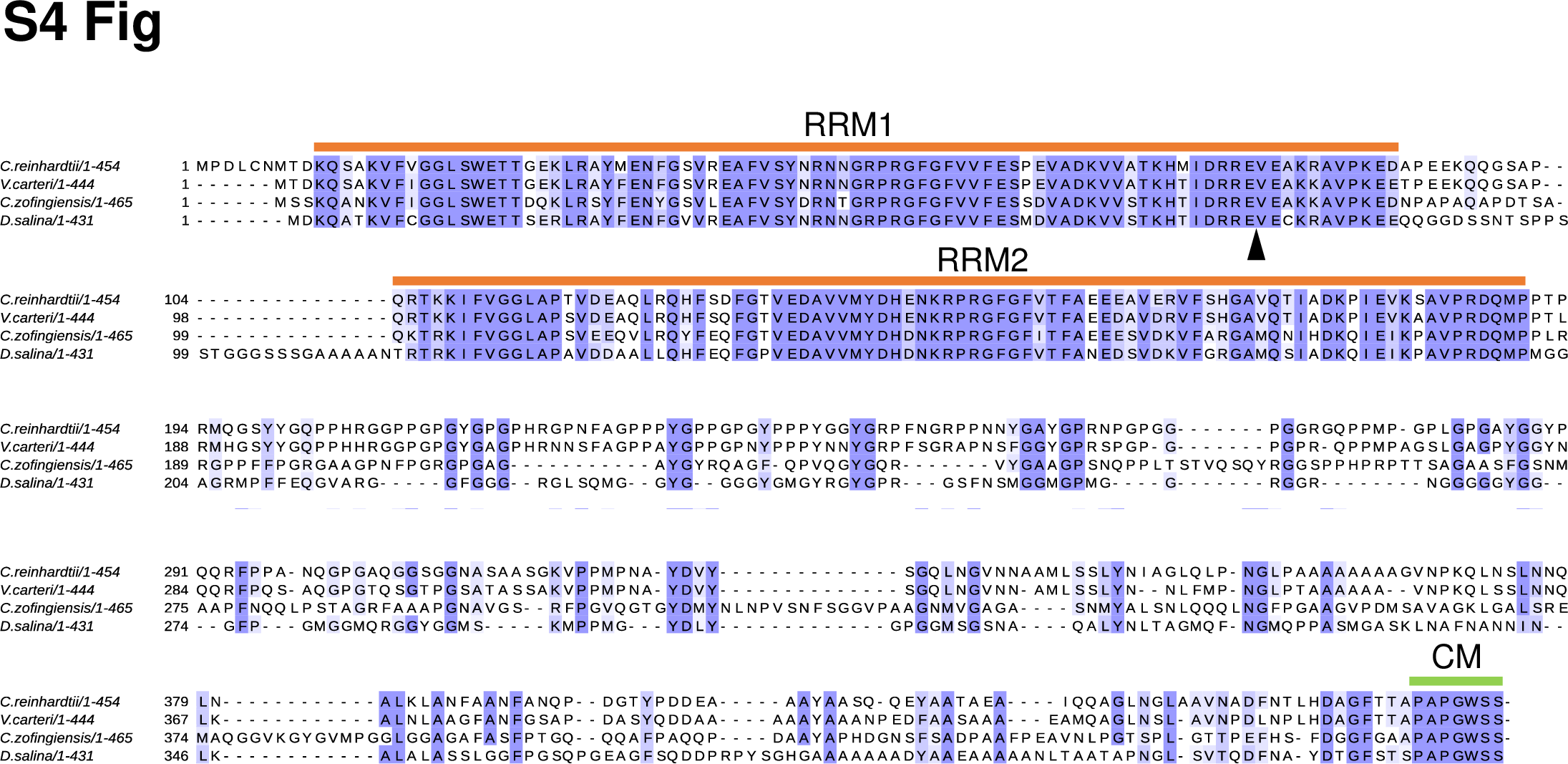

Supplement: S4 Fig — Peptide alignments for subset of proteins from Fig 2: Chlamydomonas reinhardtii TNY1 (Cre07.g330300), Volvox carteri (Vocar.0031s0001), Chromochloris zofingiensis (Cz12g11070), and Dunaliella salina (Dusal.0065s00006). Gene IDs are from Phytozome [16]. Alignment is shaded to show conserved residues. Positions of RNA recognition motifs 1 and 2 (RRM1, RRM2) and a conserved C-terminal motif (CM) are marked. The inverted black triangle shows the position of the single intron found in TNY1 orthologs in the green algal subclade. (TIF) [file pgen.1010503.s004.tif]

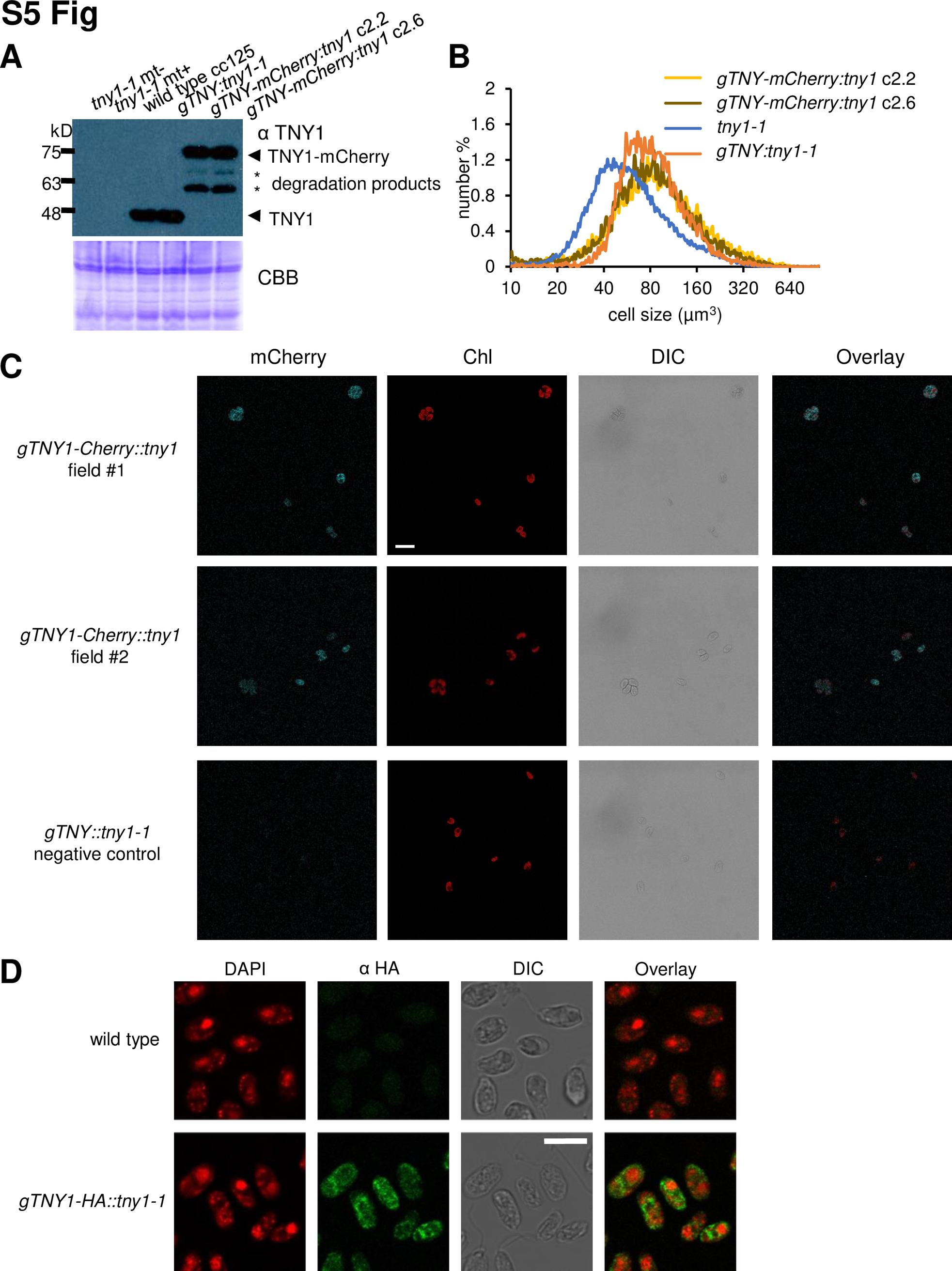

Supplement: S5 Fig — (A) Immunoblots with whole cell lysates of daughter cells from indicated genotypes. The gel was loaded with equal protein per lane, fractionated by SDS PAGE, and immunoblotted using α-TNY1 (upper panel). Coomassie blue (CBB) staining is shown in the lower panel as a loading control. (B) Size distributions of daughter cells from tny1-1 (median size 55 μm3/modal size 46 μm3), a tny1 rescue strain gTNY1:tny1-1 (median size 79 μm3/modal size 70 μm3), and two independent mCherry tagged rescue TNY1-mCherry::tny1 strains (strain c2.2 median size 81 μm3/modal size 83 μm3, strain c2.6 median size 86 μm3/modal size 79 μm3). Median sizes of TNY1-mCherry::tny1 transformants and gTNY1:tny1-1 rescued strains are not different (p>0.1, Student’s t-test) (S1 Table). (C) Fields of TNY1-mCherry::tny1 cells, along with gTNY1::tny1 cells as the negative control under the same detection settings. Annotation is the same as Fig 3. Scale bar = 20 μm. (D) DIC and immunofluorescence microscopy images of wild type CC-124 and gTNY1-HA::tny1-1. Daughter cells were fixed and immunostained for HA epitope (pseudo-colored green). DNA was stained with DAPI (pseudo-colored red). Merged fluorescence images (Overlay). Scale bar = 10 μm. (TIF) [file pgen.1010503.s005.tif]

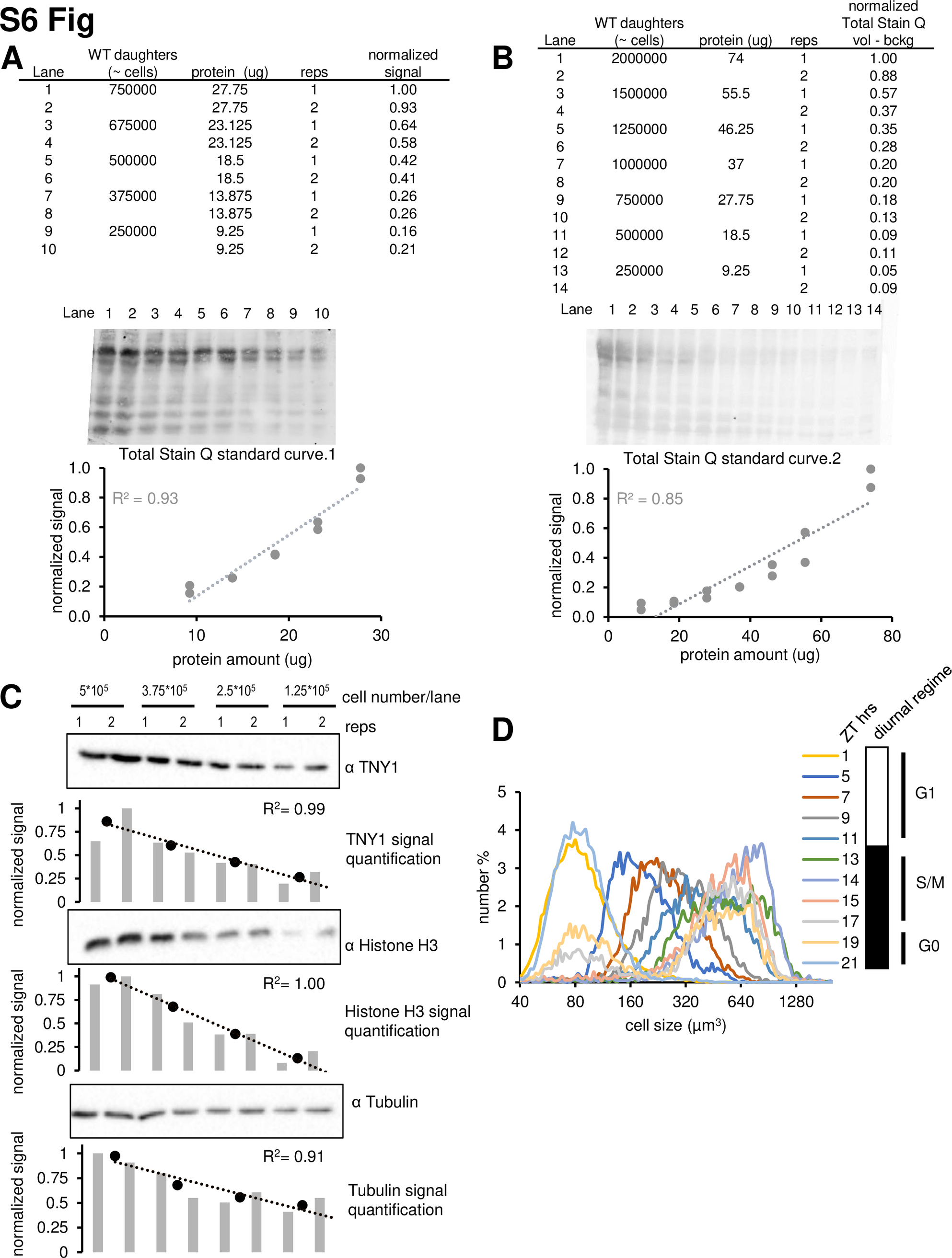

Supplement: S6 Fig — (A) and (B) Protein lysates were made using indicated cell numbers of wild-type daughter cells. Protein quantity was determined using a standard BCA kit with two replicates for each standard concentration. TotalStain Q staining signal across a range of loading amounts are documented with two replicates per sample. The band of the highest signal was set to be 1 in each blot. (A) Protein loading range used for most experiments with 9–30 μg/lane. (B) Expanded total protein loading dilution series with 9–75 μg/lane. The grayscale images were of the longest exposures without any saturated pixels. Linear regression lines (grey) are plotted for each data series. (C) Immunoblots with protein lysates made using indicated cell numbers of wild type daughter cells. Over the normal protein loading range the signals of α-TNY1, α-Histone H3, α-Tubulin are approximately linear. The band with the highest signal was set to 1 in each plot. Two independent replicates were plotted side by side (rep1 and rep2) with linear regression plotted from the average of the two repeats (black dots) for each antibody. (D) Representative size distributions of a synchronous wild type strain CC-124 at different ZT time points throughout a standard 12hr:12hr light:dark cycle. Protein lysates at each ZT were collected for immunoblots. (TIF) [file pgen.1010503.s006.tif]

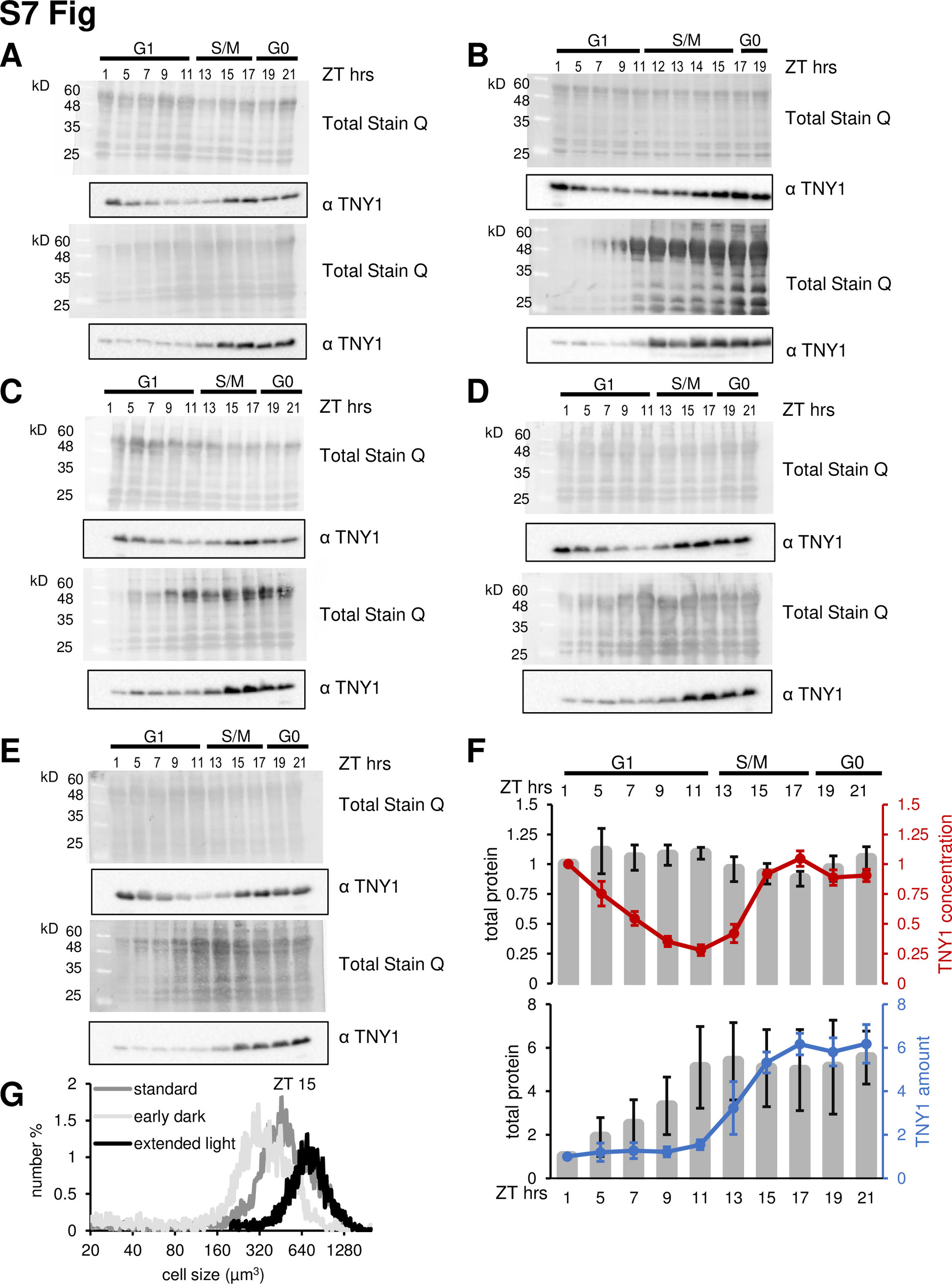

Supplement: S7 Fig — (A)–(E) Immunoblot repeats as described in Fig 4B. Three biological replicate sets with two technical repeats are included as follows: biological replicate set 1—S7A and S7B Fig; biological replicate set 2—Figs 4B and S7C; biological replicate set 3—S7D and S7E Fig. (F) Data were plotted as in Fig 4C with the inclusion of total protein (grey bars) from TotalStain Q staining with the ZT1 value set to 1. Bar values/dots represent the average of three biological repeat sets with two technical replicates each. Error bars: standard deviation of three biological replicates. (G) Size distributions of mitotic populations at ZT 15 under different diurnal regimes in Fig 4A. Standard regime at ZT 15, mean cell size 511 μm3. Early dark regime at ZT 15, mean cell size 341 μm3. Extended light regime at ZT 15, mean cell size 581 μm3. (TIF) [file pgen.1010503.s007.tif]

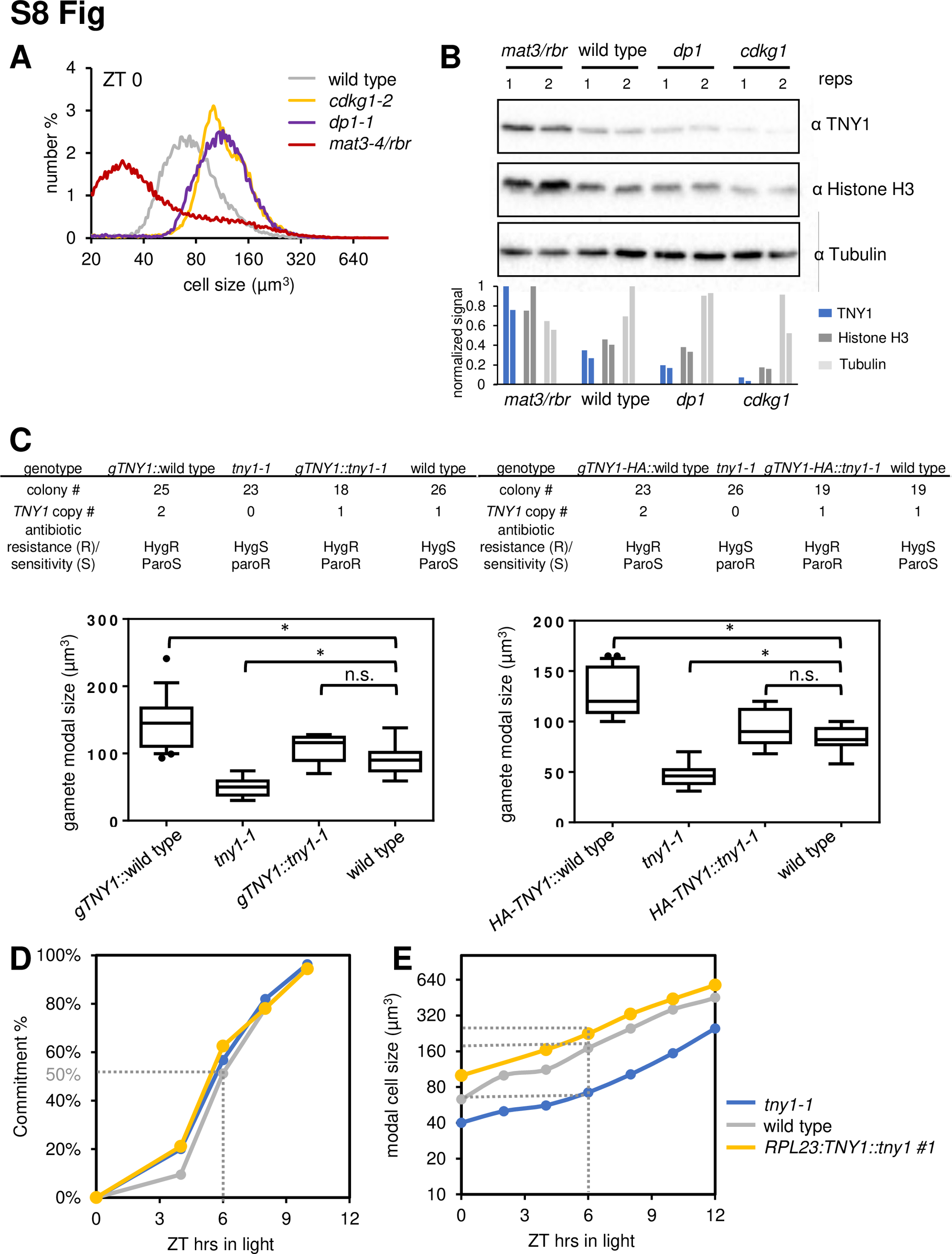

Supplement: S8 Fig — (A) Daughter size distributions (ZT 0 equivalent) of dark-shifted size mutants compared with a wild-type strain. mat3-4/rbr (median size 37 μm3/modal size 28 μm3), wild type (median size 73 μm3/modal size 75 μm3), dp1-1 (median size 111 μm3/modal size 123 μm3), and cdkg1-2 (median size 114 μm3/modal size 108 μm3). (B) Immunoblot of samples in Fig 5A with gel loading by equal protein per lane with signal quantitation shown in bar plots below. Annotation is the same as Fig 5A. (C) Left panel, box and whiskers plots of modal gamete sizes of populations derived from a back-cross between wild type CC124 and rescued strains gTNY::tny1-1 (left side) or gTNY::tny1-1 (right side). Each data point represents the modal size of a gamete population derived from an independent meiotic progeny. Numbers of progeny for each genotype sampled are listed in the table above each plot. Boxes enclose the second quartile of data with horizontal lines showing median values, and whiskers enclose the 10th - 90th percentiles. Outliers are plotted as individual data points. Comparisons among the four genotypes were done using a one-way ANOVA with post-hoc Tukey HSD Test. *, samples are different at p < 0.01; n.s., samples are not significantly different (p>0.05). (D) Plot showing timing of passing Commitment for indicated genotypes, similar to S1A Fig. Grey dotted lines mark Commitment timing of tny1-1, wild type, and a RPL23:TNY tny1-1 strain with a large size phenotype. Plot of modal cell sizes for cultures in panel (D). Grey dotted lines mark cell sizes of strains in panel (E) showing that tny1-1, wild type, and the RPL23:TNY tny1-1 strain pass Commitment at about the same ZT. Commitment sizes for each genotype: tny1 ~ 80 μm3, wild type ~ 200 μm3, RPL23:TNY tny1-1 #1 ~ 250μm3. (TIF) [file pgen.1010503.s008.tif]

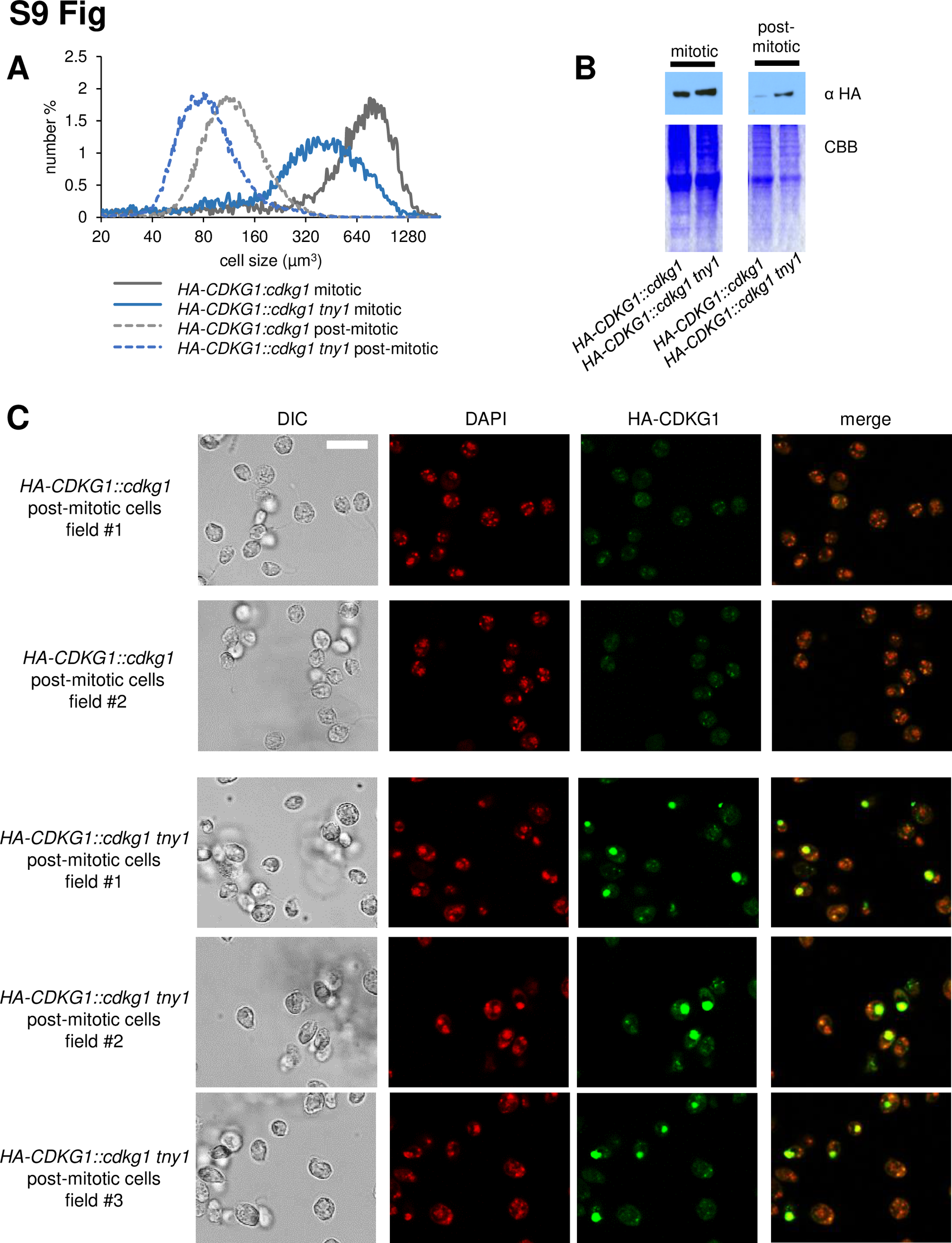

Supplement: S9 Fig — (A) Size distributions of synchronous mitotic (ZT 13) and post-mitotic (ZT 1) populations of indicated strains. (B) Immunoblots using synchronized strains of indicated genotypes loaded with equal numbers of cells per lane and probed with α-HA to detect HA-CDKG1 or stained with Coomassie blue (CBB). (C) Immunofluorescence images of HA-CDKG1::cdkg1 and HA-CDKG1::cdkg1 tny1 post-mitotic cells (ZT 1) as described in Fig 6C. Scale bar = 10 μm. (TIF) [file pgen.1010503.s009.tif]

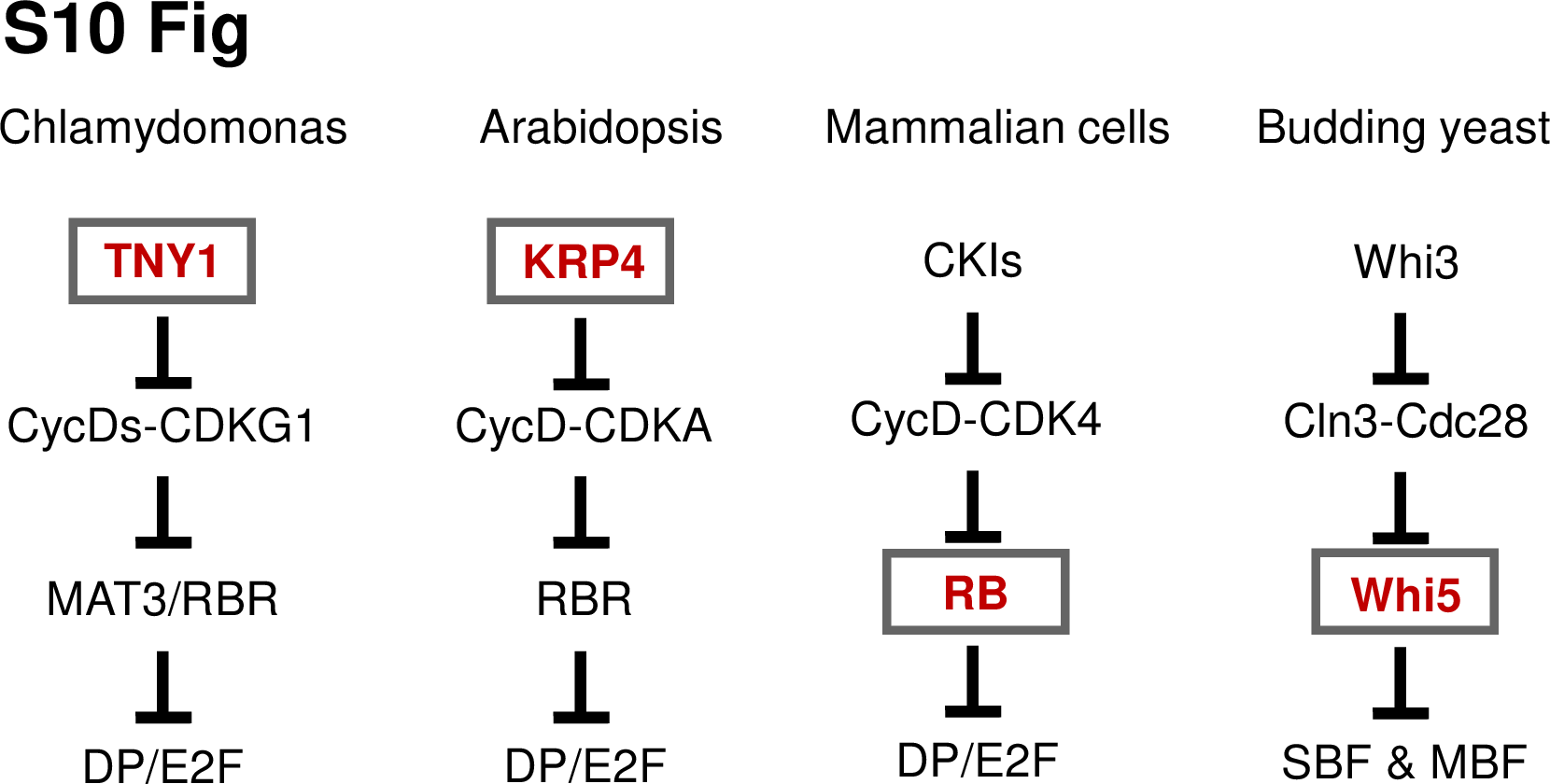

Supplement: S10 Fig — Cell cycle inhibitors subscaling with cell size in G1 phase are highlighted in bold red. (TIF) [file pgen.1010503.s010.tif]
